# miR‐486 improves fibrotic activity in myocardial infarction by targeting SRSF3/p21‐Mediated cardiac myofibroblast senescence

**DOI:** 10.1111/jcmm.17539

**Published:** 2022-09-18

**Authors:** Hongyi Chen, Luocheng Lv, Ruoxu Liang, Weimin Guo, Zhaofu Liao, Yilin Chen, Kuikui Zhu, Ruijin Huang, Hui Zhao, Qin Pu, Ziqiang Yuan, Zhaohua Zeng, Xin Zheng, Shanshan Feng, Xufeng Qi, Dongqing Cai

**Affiliations:** ^1^ Key Laboratory of Regenerative Medicine, Ministry of Education Jinan University Guangzhou China; ^2^ Joint Laboratory for Regenerative Medicine Chinese University of Hong Kong‐Jinan University Guangzhou China; ^3^ International Base of Collaboration for Science and Technology (JNU) Ministry of Science and Technology Guangzhou China; ^4^ Department of Developmental and Regenerative Biology Jinan University Guangzhou China; ^5^ Institute of Anatomy, Department of Neuroanatomy, Medical Faculty University of Bonn Germany; ^6^ Stem Cell and Regeneration TRP, School of Biomedical Sciences Chinese University of Hong Kong Hong Kong; ^7^ Cancer Institute of New Jersey Department of Medical Oncology, Robert Wood Johnson of Medical School USA; ^8^ Division of Cardiology, Department of Internal Medicine The First Affiliated Hospital of Guangzhou Medical University Guangzhou China

**Keywords:** fibrosis, miR‐486, regeneration of myocardial infarction, senescence of cardiac myofibroblasts, SRSF3

## Abstract

The regulation of fibrotic activities is key to improving pathological remodelling post‐myocardial infarction (MI). Currently, in the clinic, safe and curative therapies for cardiac fibrosis and improvement of the pathological fibrotic environment, scar formation and pathological remodelling post‐MI are lacking. Previous studies have shown that miR‐486 is involved in the regulation of fibrosis. However, it is still unclear how miR‐486 functions in post‐MI regeneration. Here, we first demonstrated that miR‐486 targeting *SRSF3/p21* mediates the senescence of cardiac myofibroblasts to improve their fibrotic activity, which benefits the regeneration of MI by limiting scar size and post‐MI remodelling. miR‐486‐targeted silencing has high potential as a novel target to improve fibrotic activity, cardiac fibrosis and pathological remodelling.

## 1 INTRODUCTION

Myocardial infarction (MI) is the leading cause of death worldwide.^1^ Improvement of profibrotic activity and pathological remodelling in ischaemic myocardium mediated by activated cardiac myofibroblasts (CMFs) post‐MI are still major challenges. Cardiac fibroblasts (CFs) play an important role in cardiac repair, scar formation, fibrosis and pathological reconstruction post‐MI.^2^, ^3^ In MI, loss of structural integrity of the myocardium exposes CFs to mechanical stress, accompanied by particular inflammatory effectors, cytokines, hormones and growth factors, inducing CF proliferation, migration to the infarct zone and transdifferentiation into CMFs. In the adult mammalian heart, after MI, due to a very low potency to regenerate cardiomyocyte death, the dead cardiomyocytes are replaced by fibrotic tissue and scar, which is secreted by CMFs. CMFs are responsible for scar tissue formation at every site of cardiomyocyte necrosis and fibrosis in the ischaemic myocardium.^4^, ^5^ Accompanied by cardiomyocyte loss and scar formation post‐MI, the following response is remodelling of the ischaemic myocardium and eventually incurs hypertrophy and fibrosis of the left ventricular wall. This remodelling process is progressive in the post‐MI myocardium and eventually leads to heart failure.^6^ However, the mechanism of maintaining physiopathology for fibrotic and antifibrotic environments for cardiac fibrosis and remodelling in ischaemic myocardium post‐MI is still unclear.

A recent study demonstrated that *p*53‐mediated senescence of cardiac fibroblasts is crucial to limit cardiac fibrosis via downregulation of cardiac collagen production after myocardial infarction.^7^ CMF senescence is an essential requirement for antifibrotic mechanisms, and CCN1, a potent inducer of senescence, was able to reduce perivascular fibrosis by approximately 50%.^8^ More recently, it was revealed that CCN1‐induced cellular senescence of cardiac fibroblasts was able to promote heart regeneration.^9^ The up‐to‐date progress in the field proposes that cellular senescence of cardiac fibroblasts acts as an essential mechanism for antifibrosis and heart regeneration. However, the critical effectors regulating this essential mechanism to improve the fibrotic environment and cardiac regeneration, which are targeted for CFs and CMFs, are still unclear. Currently, in the clinic, safe and curative therapies for cardiac fibrosis and improvement of the pathological fibrotic environment, scar formation and pathological remodelling post‐MI are lacking.

MicroRNAs (miRNAs) are noncoding RNAs that are 19–22 nucleotides in length that negatively regulate target gene expression by interacting with the 3'‐UTR regions of mRNAs at the posttranscriptional or translational level.^10^ It has been demonstrated that miRNAs participate in a broad range of cardiac pathophysiologies and cardiovascular functions.^11^ miRNA‐21, miRNA‐29 and miRNA‐34 have been reported to play an important role in the regulation of cardiac fibrosis. Downregulation of miRNA‐21^12^ and miRNA‐34^13^ reduced fibrosis, while silencing of miRNA‐29 exacerbated the production of collagen.^14^ Furthermore, recent studies have demonstrated that miR‐486‐5*p* plays an important regulatory role in the inhibition of fibroblast proliferation in hypertrophic scars.^15^, ^16^ In addition, miR‐486‐5*p* was shown to be involved in the cellular senescence of human diploid fibroblasts^17^ and IgE elevation‐mediated pathologic cardiac fibrosis.^18^ These findings suggest that miR‐486 might be an important endogenous modulator of the antifibrotic environment in fibroblasts and myofibroblasts. However, it is still unclear how miR‐486 functions in post‐MI regeneration. Accordingly, we hypothesized that miR‐486 might act as an important player in suppressing fibrotic activity in MI, which benefits the improvement of cardiac fibrosis, pathological remodelling and regeneration of MI. Indeed, in the present study, we first demonstrated that miR‐486 targeting *SRSF3/p21* mediates the senescence of cardiac myofibroblasts to improve their fibrotic activity, which benefits the improvement of post‐MI cardiac fibrosis, pathological remodelling and regeneration.

## 2 MATERIAL AND METHODS

### 2.1 Animals

In present study, two‐month‐old female Sprague–Dawley (SD) rats (200–250 g) were used. The rats were adapted for 1 week feeding before experimentation. The animals were provided with food and water ad libitum. Animal care, surgery and handling procedures were approved by the Jinan University Animal Care Committee (Approval No. IACUC‐20190104‐010).

### 2.2 Preparation and culture of CMFs

CFs were isolated from the ventricles of 2‐month‐old female SD rats as published reported.^19^ For details, see Appendix.

### 2.3 Immunofluorescence of Vimentin, α‐smooth muscle Actin, Cardiac Troponin I, CD68, CD163 and CD11b

For details, see Appendix.

### 2.4 Isolation of total RNA and real‐time quantitative PCR

TRIzol reagent (cat. no. 15596018; Invitrogen) was used to extract Total RNA. The Hairpin‐it miRNA q‐PCR Quantitation Kit (cat. no. E01006‐E01015; GenePharma) was applied to quantify rno‐miR‐486 expression. For details, see Appendix.

### 2.5 Transfection of rno‐miR‐486 mimics in CMFs

The isolated CMFs were transfected with rno‐miR‐486 mimics (5'‐UCCUGUACUGAGCUGCCCCGAG‐3′; 100 nM; GenePharma). For details, see Appendix.

### 2.6 β‐galactosidase staining

The prepared cells or frozen sections were used for senescence β‐gal staining using a β‐galactosidase staining kit (C0602; Beyotime). For details, see Appendix.

### 2.7 β‐galactosidase fluorescence assay

After prepared CMFs were treated with miR‐486 for 48 hours, cellular senescence was analysed using the ImaGene Green™ C_12_FDG lacZ Gene Expression Kit (I‐2904; Invitrogen). For details, see Appendix.

### 2.8 Cell proliferation assay

A CCK‐8 assay (CK04; Dojindo) was performed to analyse the proliferation of CMFs after miR‐486 treatment. For details, see Appendix.

### 2.9 Apoptosis assay

The Annexin Alexa Fluor 488/PI kit (FXP022‐100, 4A Biotech) and subsequent flow cytometry analysis were applied to investigate the apoptosis of miR‐486 treated CMFs. For details, see Appendix.

### 2.10 Cell cycle assay

PI/RNase Staining Buffer (550,825; BD) was applied to analyse the cell cycle of CMFs after miR‐486 treatment for 72 hours. For details, see Appendix.

### 2.11 Western blot analysis

The radioimmunoprecipitation assay (RIPA) buffer (cat. no. P0013C; Beyotime) containing a protease inhibitor cocktail (cat. no. W2200s; Cwbio) was applied to prepare the CMFs lysates. Please note that the Western blot band of GAPDH in Figure 2B‐B1 and Figure 3C‐C1, serving as the internal control, is the same image, as the two figures were obtained from the same piece of gel for electrophoresis separation, membrane transfer and subsequent analysis. The same experimental setting for GAPDH was also for Figure 4B‐B1 and Figure 4E‐E1. For details, see Appendix.

### 2.12 Prediction of miR‐486 targeted genes

TargetScan (http://www.targetscan.org) was used to predict the potential target genes of rno‐miR‐486. FirePlex Discovery Engine (https://www.fireflybio.com/ portal/search) was used to verify whether or not a gene is already reported as target gene for miR‐486.

### 2.13 Dual‐luciferase reporter assay

Luciferase reporters were constructed by cloning sequences from the 3' untranslated regions (3'UTRs) of the *SRSF3* mRNAs into the psiCHECK‐2 vector (cat. No. C8021, Promega). For details, see Appendix.

### 2.14 RNA interference

The synthesized si*SRSF3* (5'‐CCCAAGAAGGAGAAGCTTT‐3') and siRNA negative control (RIBOBIO, China) were transfected into CMFs using Lipofectamine RNAiMAX (cat. No. 13778150; Invitrogen). For details, see Appendix.

### 2.15 RIP‐Qpcr

The prepared CMFs were cultured to approximately 80% confluency, and then, 2 × 10^6^ cells were collected for RIP‐qPCR assay. An Imprint® RNA Immunoprecipitation Kit (cat. no. RIP‐12RXN; Sigma) was used. For details, see Appendix.

### 2.16 Induction of MI and intramyocardial injection

Two‐month‐old female SD rats were applied to establish MI. For details, see Appendix.

### 2.17 Echocardiography

Transthoracic echocardiograms were performed to analyse the cardiac function in the experimental rats. The ejection fraction (EF) was calculated using the area‐length method.^20^ For details, see Appendix.

### 2.18 Histological analysis

The MI extent was analysed at the level of the mid‐papillary heart muscles and scored following Masson's trichrome staining. The infarct size, with linear approximations to account for area gaps in histology, was expressed as a percentage of the total LV myocardial area as our and others previously described.^20^, ^21^ The area of cardiomyocytes was evaluated by Wheat Germ Agglutinin (WGA) staining. For details, see Appendix.

### 2.19 Immunohistochemistry staining

The immunostaining for vWF, a marker of endothelial cells, was applied to measure small blood vessel density in the infarct zone and border zone. For details, see Appendix.

### 2.20 Treadmill test

A treadmill (SA101C, Sansbio) was used to measure the endurance and physical fitness. For details, see Appendix.

### 2.21 Statistical analysis

An independent samples *t*‐test was performed using GraphPad prism software to determine the P‐values in repeated experiments. All values are expressed as the mean ± standard deviation (S.Dev). *p* < 0.05 was set as statistically significant differences.

## 3 RESULTS

### 3.1 miR‐486 promotes cellular senescence and apoptosis and inhibits proliferation in CMFs in vitro

**FIGURE 1:**
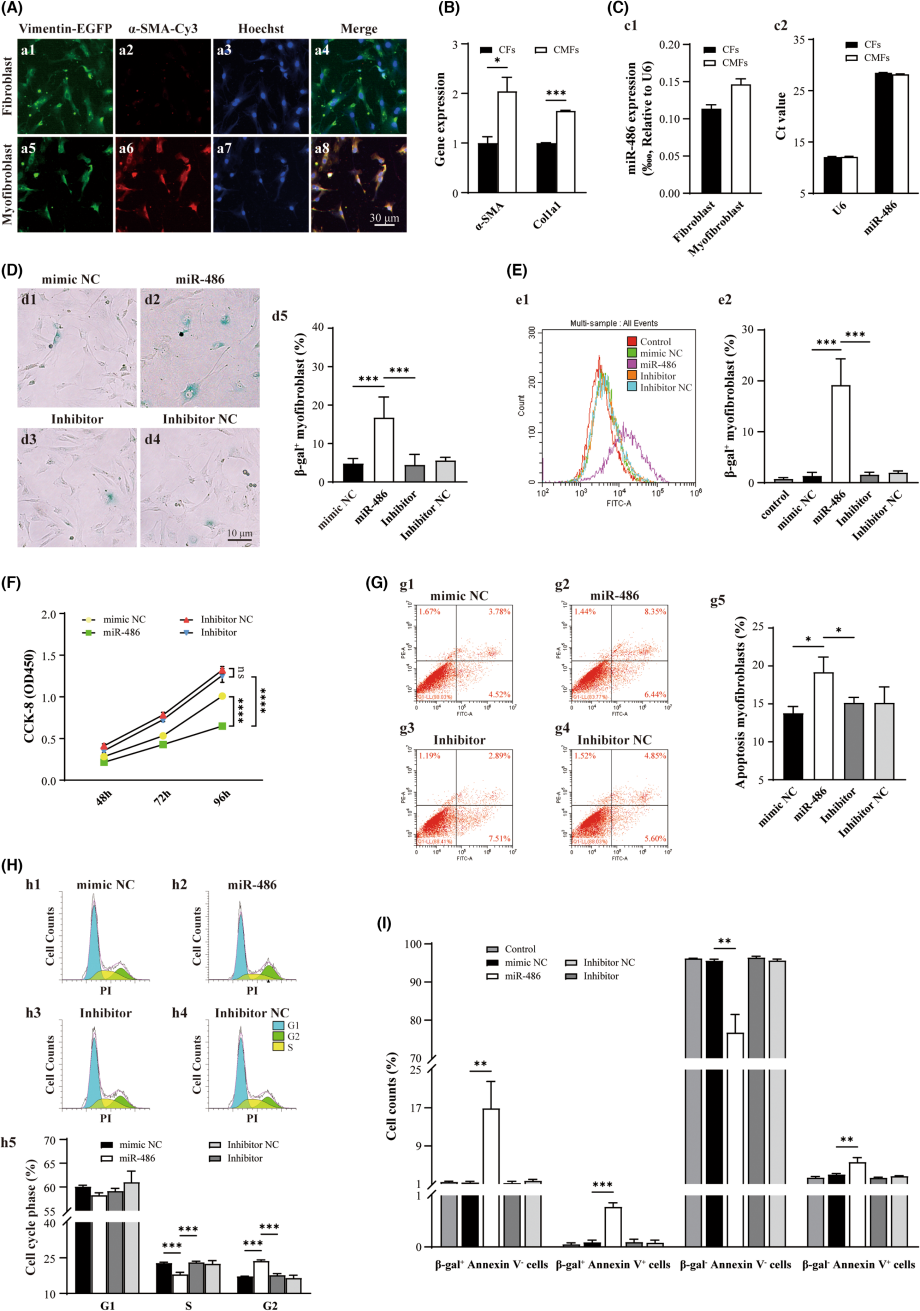
miR‐486 promotes cellular senescence and apoptosis and inhibits proliferation and the cell cycle at the S and G2/M phases in CMFs in vitro. (A) Immunofluorescence images of isolated CFs and TGF‐β‐induced transdifferentiation CMFs (Vimentin‐EGFP, α‐SMA‐Cy3). (B) The expression of *α‐SMA* and *Col1a1* was significantly increased in CMFs, which confirmed successful transdifferentiation to CMFs. (C) miR‐486 is expressed in both CFs and CMFs. (C1) and (C2) The expression level of miR‐486 in CFs and CMFs is shown as a ratio to the U6 and Ct values. (D) β‐gal histological staining showed that the number of β‐gal‐positive CMFs was increased significantly in the miR‐486‐treated group. (D1‐4) Representative images of the mimic NC‐, miR‐486‐, inhibitor‐ and inhibitor NC‐treated groups. (D5) Quantitative analysis of (D1‐4). (E) The flow cytometry quantitative assay double confirmed that the number of β‐gal‐positive CMFs was increased significantly in the miR‐486‐treated group. (E1) Representative pattern of flow cytometry analysis. (E2) Quantitative analysis of (E1). (F) The CCK‐8 assay showed that the proliferation of CMFs in the miR‐486‐treated group was significantly lower than that in the mimic NC and control groups. (G) Flow cytometry analysis of apoptosis showed that the number of apoptotic cells in the miR‐486‐treated CMFs was significantly higher than that in the mimic NC‐treated CMFs. (G1‐4) Representative patterns of the mimic NC‐, miR‐486‐, inhibitor‐ and inhibitor NC‐treated groups. (G5) Quantitative analysis of (G1‐4). (H) Cell cycle analysis with flow cytometry revealed that miR‐486 overexpression in CMFs was able to promote cell cycle inhibition at the S and G2/M phases. (H1‐4) Representative patterns of the mimic NC‐, miR‐486‐, inhibitor‐ and inhibitor NC‐treated groups. (H5) Quantitative analysis of (H1‐4). (I) The double staining analysis of cellular senescence and apoptosis in miR‐486‐treated CMFs suggests that in vitro, miR‐486 overexpression is able to promote cellular senescence and apoptosis of CMFs and induce more senescent CMFs than apoptotic CMFs. Importantly, most miR‐486‐treated senescent CMFs do not undergo apoptosis simultaneously. *n* = 3.

To investigate the functional role of miR‐486 in cardiac myofibroblasts (CMFs), CMFs were generated by TGF‐β‐induced transdifferentiation from cardiac fibroblasts (Vimentin^+^; CFs), which were isolated from rat ventricles. The transdifferentiated CMFs were confirmed by the expression of the *α‐SMA* and *Col1a1* genes and immunofluorescence of Vimentin‐EGFP and α‐SMA‐Cy3 (Figure 1A, B; *p* < 0.05). The results showed that miR‐486 was expressed in both CFs and CMFs (Figure 1C). Furthermore, overexpression of miR‐486 in CMFs was able to promote cellular senescence, which was confirmed by a significant increase in the density of β‐gal‐positive cells in the miR‐486‐treated group compared to the mimic NC‐treated group via β‐gal histological staining and cytometry quantitative assays (Figure 1D, E; *p* < 0.05). It was revealed that miR‐486 overexpression mediated CMF senescence in a dose‐dependent manner, and 100 nmol/L was a suitable dose (Figure S1A). Therefore, 100 nmol/L miR‐486 was applied in the in vitro study. In addition, the CCK‐8 assay indicated that the proliferation and survival rates of the miR‐486‐treated CMFs were significantly lower than those of the mimic NC‐treated CMFs (Figure 1F; *p* < 0.05). Flow cytometry analysis of apoptosis showed that the number of apoptotic cells in the miR‐486‐treated CMFs was significantly higher than that in the mimic NC‐treated CMFs (Figure 1G; *p* < 0.05) in a dose‐dependent manner (Figure S1B). Furthermore, flow cytometry analysis of the cell cycle showed that overexpression of miR‐486 in CMFs was able to promote cell cycle inhibition at the S and G2/M phases (Figure 1H; *p* < 0.05). Double staining analysis of cellular senescence and apoptosis in miR‐486‐treated CMFs was further performed to determine whether miR‐486 overexpression in CMFs mediates both senescence and apoptosis simultaneously. It was found that miR‐486‐mediated cellular senescence and apoptosis coexisting in cells (β‐gal‐positive and Annexin V‐positive cells) was rare (an increase of only 0.69% vs. mimic NC). However, miR‐486‐mediated senescent cells (β‐gal‐positive cells and Annexin V‐negative cells) showed a 15.58% increase (vs. mimic NC), and miR‐486‐mediated apoptotic cells (β‐gal‐negative cells and Annexin V‐positive cells) showed a 2.61% increase (vs. mimic NC) (Figure 1I). Taken together, these findings show that in vitro, miR‐486 overexpression is able to promote cellular senescence and apoptosis of CMFs and induce more senescent CMFs than apoptotic CMFs. Importantly, most miR‐486‐treated senescent CMFs do not undergo apoptosis simultaneously. In addition, miR‐486 overexpression inhibited proliferation by inhibiting the cell cycle at the S and G2/M phases in CMFs.

### 3.2 miR‐486 activates the *p*21 cellular senescence pathway and inhibits the expression of fibrotic genes in CMFs in vitro

**FIGURE 2:**
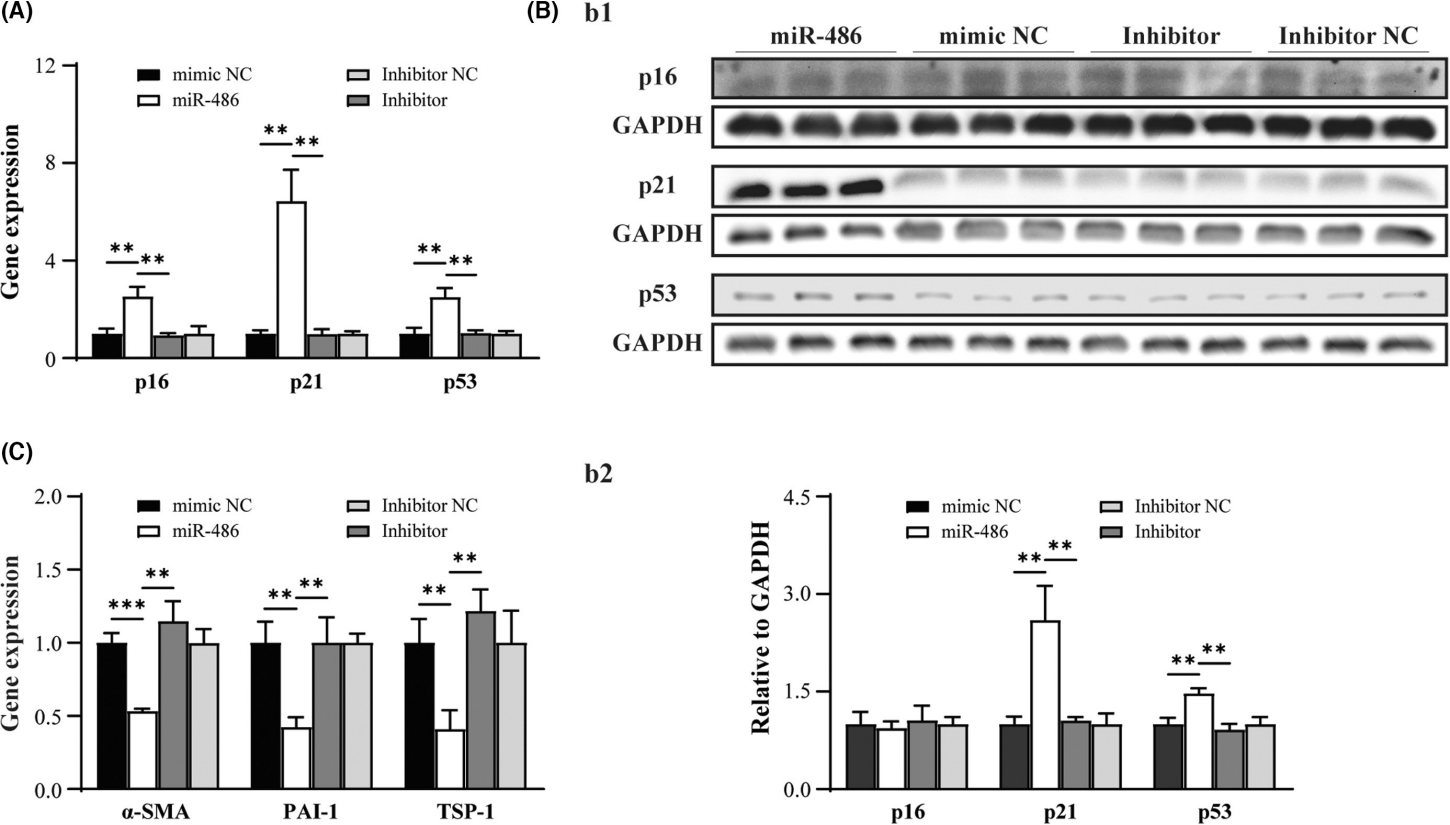
miR‐486 activates the *p*21 cellular senescence pathway and inhibits the expression of fibrosis effector genes in CMFs in vitro. (A) The qPCR results showed that miR‐486 overexpression in CMFs increased the mRNA expression of the senescence‐regulating genes *p21*, *p53* and *p16*. (B) Western blot confirmed that miR‐486 overexpression in CMFs increased *p*21 and *p*53 expression at the protein level. B1: Representative image of Western blot. (B2) Quantitative analysis of b1. (C) The qPCR results showed that miR‐486 overexpression in CMFs decreased the expression of fibrosis effector genes (*PAI‐1*, *TSP‐1* and *α‐SMA*). *n* = 3.

The pathway and functional effects related to miR‐486‐mediated cellular senescence in CMFs were investigated. The qPCR results revealed that miR‐486 overexpression in CMFs increased the expression of the well‐recognized cellular senescence‐regulating genes *p21*, *p53* and *p16* at the mRNA level (Figure 2A; *p* < 0.05). However, only the *p*21 and *p*53 protein levels were increased (Figure 2B; *p* < 0.05). In addition, the expression of well‐established fibrosis effector genes (plasminogen activator inhibitor‐1: *PAI‐1*, thrombospondin 1: *TSP‐1* and *α‐SMA*)^3^, ^22^, ^23^, ^24^, ^25^, ^26^ in the miR‐486‐treated CMFs was significantly decreased compared with that in the mimic NC‐treated CMFs (Figure 2C; *p* < 0.05). The findings suggested that overexpression of miR‐486 is able to trigger the cellular senescence of CMFs via activation of the *p*53/*p*21 pathway and inhibit fibrotic potency in CMFs in vitro.

### 3.3 miR‐486‐targeted silencing of *SRSF3* expression induces *p*21 activation‐mediated cellular senescence in CMFs

**FIGURE 3:**
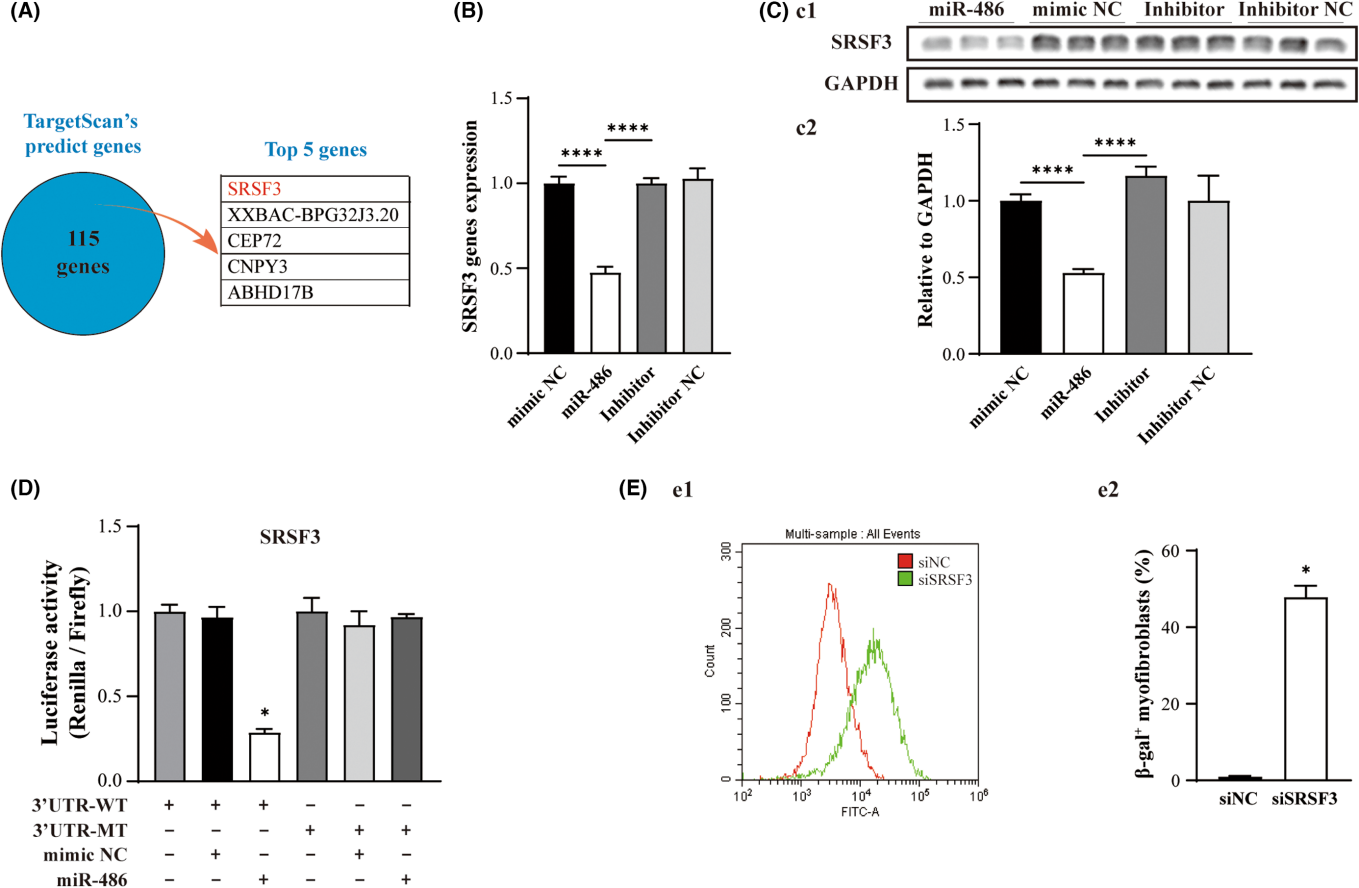
SRSF3 is a target gene of miR‐486, and *SRSF3* silencing promotes cellular senescence of CMFs in vitro. (A) The top five potential target genes were predicted by the TargetScan database. (B) Gene expression of *SRSF3* was significantly decreased in miR‐486‐treated CMFs, as shown by qPCR. (C) Transfection of miR‐486 into CMFs decreased SRSF3 protein expression. (D) The results of the dual‐luciferase assay showed that *SRSF3*‐3'UTR was able to interact with miR‐486. (E) The β‐gal fluorescent flow cytometry quantitative assay showed that the density of β‐gal‐positive CMFs in the si*SRSF3*‐treated group was significantly higher than that in the siNC‐treated group. (E1) Representative pattern of flow cytometry analysis. (E2) Quantitative analysis of e1. *n* = 3

The mechanism by which miR‐486 promotes cell senescence via activation of *p*21 was further investigated. A total of 115 potential target genes were predicted by the TargetScan database. The top five predicted potential target genes of miR‐486 by TargetScan are listed in Figure 3A. Among them, *SRSF3*, an effector of mRNA alternative splicing,^27^, ^28^, ^29^ was selected for further investigation because it ranked first and was reported to be involved in cellular senescence regulation,^27^, ^28^ and it has not yet been demonstrated as a target gene of miR‐486 in the literature. We measured the mRNA and protein levels using qPCR and Western blotting and found that the expression of SRSF3 was decreased significantly in miR‐486‐treated CMFs (Figure 3B, C; *p* < 0.05). In addition, the results of the dual‐luciferase assay showed that *SRSF3* can be regulated by miR‐486 through its 3'UTR and confirmed that *SRSF3* is a novel target gene of miR‐486 (Figure 3D) that can be silenced by miR‐486 (Figure 3B, C). Furthermore, the effect of *SRSF3* on cellular senescence was investigated and showed that *SRSF3* silencing was able to significantly increase the density of β‐gal‐positive cells (Figure 3E; *p* < 0.05).

**FIGURE 4:**
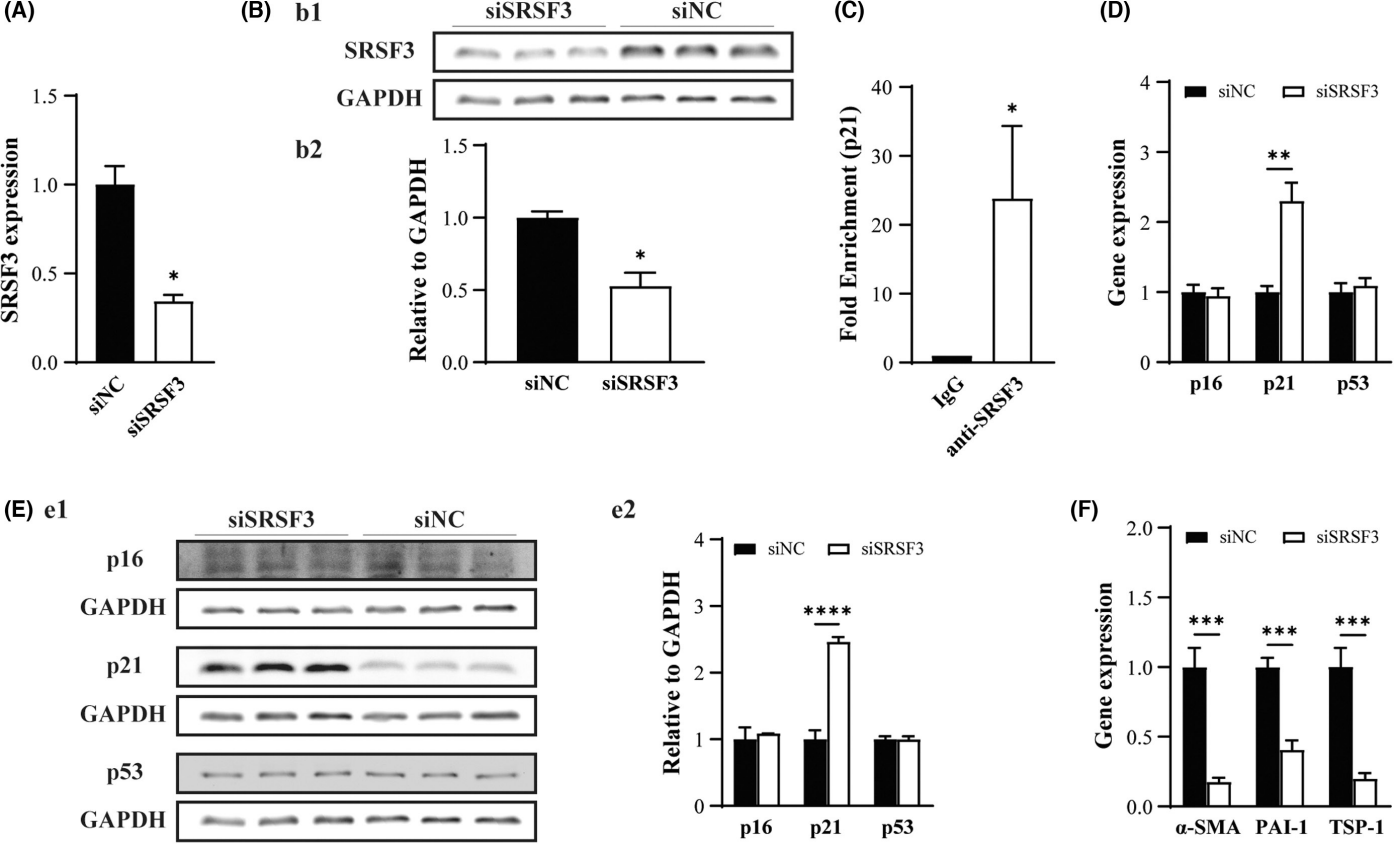
Silencing *SRSF3* expression induces *p*21 activation accompanied by a decrease in the expression of fibrosis effector genes in CMFs in vitro. (A‐B) Transfection of si*SRSF3* in CMFs significantly downregulated the expression of SRSF3 at the mRNA level (A) and protein level (B). (B1) Representative pattern of Western blot for si*SRSF3*‐ and siNC‐treated group. (B2) Quantitative analysis of (B1). C: RIP‐qPCR revealed that SRSF3 was able to bind with *p21* in CMFs. (D‐E) Transfection of si*SRSF3* in CMFs upregulated the expression of *p*21 significantly at the mRNA level (D) and protein level (E) but not *p*16 and *p*53. (E1) Representative pattern of Western blot for si*SRSF3*‐ and siNC‐treated group. (E2) Quantitative analysis of E1. F: *SRSF3* silencing in CMFs decreased the expression of fibrosis effector genes (*PAI‐1*, *TSP‐1* and *α‐SMA*). *n* = 3

The possible effects of the *SRSF3* gene on *p21* expression and fibrosis effector genes in CMFs were further investigated. We first confirmed that transfection of si*SRSF3* in CMFs significantly downregulated the expression of SRSF3 at both the mRNA and protein levels (Figure 4A, B). Therefore, the effect of SRSF3 silencing was next studied. We first conducted RIP‐qPCR assays to evaluate the binding capacity between SRSF3 and *p21*. The results of anti‐SRSF3 antibody‐RIP‐qPCR revealed that SRSF3 was able to bind with *p21* in CMFs (Figure 4C). Furthermore, *p*21 expression in si*SRSF3*‐treated CMFs at the mRNA and protein levels was significantly upregulated compared to that in siNC‐treated CMFs, but p16 and *p*53 were not (Figure 4D, E). In addition, the expression of fibrosis effector genes (*PAI‐1*, *TSP‐1 and α‐SMA*) in the si*SRSF3*‐treated CMFs was decreased significantly compared with that in the siNC‐treated CMFs (Figure 4F; *p* < 0.05). The above results demonstrated that knock down of *SRSF3* expression promotes cellular senescence, upregulates *p*21 expression and downregulates the expression of fibrosis effector genes (*PAI‐1*, *TSP‐1 and α‐SMA*) in CMFs.

Taken together, the results verify that in CMFs, miR‐486 targets and silences SRSF3. SRSF3 inhibits *p21* gene expression through a direct binding mechanism. Therefore, miR‐486‐targeted silencing of SRSF3 leads to *p*21 activation of cellular senescence, resulting in downregulation of the expression of fibrosis effector genes in CMFs.

### 3.4 miR‐486 expression is downregulated in the infarct zone, and its overexpression improves fibrotic pathology, fibrosis and pathological remodelling in post‐MI hearts, which benefits the regeneration of MI in vivo

**FIGURE 5:**
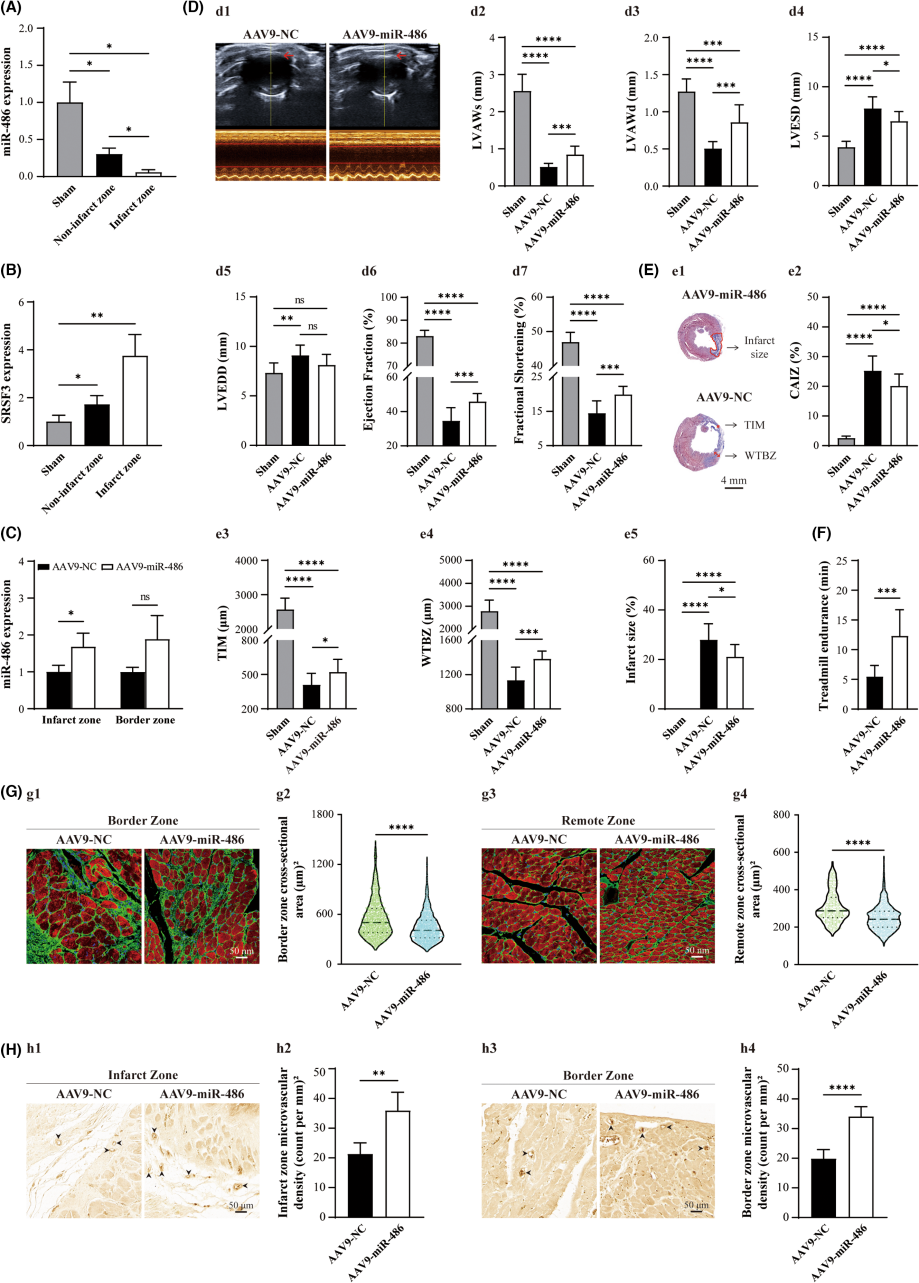
miR‐486 expression is downregulated and SRSF3 expression is upregulated significantly in the infarct zone post‐MI, and AAV9‐miR‐486‐mediated overexpression in MI improves fibrotic pathology, fibrosis and pathological remodelling in 8‐week post‐MI hearts, which benefits the regeneration of MI. (A) miR‐486 expression was downregulated significantly in both the infarct zone and noninfarct zone. *n* = 3, 6 and 6 for the sham, noninfarct zone and infarct zone groups. (B) *SRSF3* expression was upregulated significantly in both the infarct zone and noninfarct zone. *n* = 3, 5 and 5 for the sham, noninfarct zone and infarct zone groups. (C) The expression level of miR‐486 was significantly higher in the infarct zone of the AAV9‐miR‐486‐EGFP‐treated myocardium than in the AAV9‐NC‐treated myocardium. *n* = 3. (D) The echocardiography analysis (D1) showed that the left ventricular anterior wall in the end‐systolic phase (LVAWs, D2) and the left ventricular anterior wall in the end‐diastolic phase (LVAWd, D3) of the AAV9‐miR‐486‐EGFP‐treated group were significantly thicker than those of the AAV9‐NC‐treated group. The left ventricular end‐systolic diameter (LVESD, D4) of the AAV9‐miR‐486‐treated group was significantly smaller than that of the AAV9‐NC‐treated group, but the difference in the left ventricular end‐diastolic diameter (LVEDD, D5) between the AAV9‐miR‐486‐EGFP‐treated group and the AAV9‐NC‐treated group was not statistically significant. The ejection fraction (EF, D6) and fractional shortening (FS, D7) of the AAV9‐miR‐486‐EGFP‐treated group were significantly higher than those of the AAV9‐NC‐treated group. *n* = 10. (E) The analysis of Masson's trichrome staining (E1) showed that the collagen area of the infarct zone (CAIZ) in the AAV9‐miR‐486‐EGFP‐treated group was significantly smaller than that of the AAV9‐NC‐treated group (E2). The thickness of the infarcted myocardium of the left ventricle (TIM, E3) and the wall thickness of the border zone of the left ventricle (WTBZ, E4) of the AAV9‐miR‐486‐EGFP‐treated group were significantly larger than those of the AAV9‐NC‐treated group. The infarct size of the AAV9‐miR‐486‐EGFP‐treated group was significantly smaller than that of the AAV9‐NC‐treated group (E5). *n* = 10. (F) The treadmill test revealed that the running endurance time of the AAV9‐miR‐486‐EGFP‐treated group was significantly longer than that of the AAV9‐NC‐treated group. *n* = 8 and 10 for the AAV9‐NC and AAV9‐miR‐486 groups. (G) WGA staining of the cardiomyocyte area showed that the cardiomyocyte area of the AAV9‐miR‐486‐EGFP‐treated group was significantly smaller than that of the AAV9‐NC‐treated group in both the infarct zone and border zone. (G1) Representative images of the AAV9‐miR‐486‐EGFP‐ and AAV9‐NC‐treated groups in the border zone. (G2) Quantitative analysis of (G1). (G3**)** Representative images of the AAV9‐miR‐486‐EGFP‐ and AAV9‐NC‐treated groups in the remote zone. **(**G4) Quantitative analysis of G3. *n* = 8 and 10 for the AAV9‐NC and AAV9‐miR‐486 groups. (H) The results of anti‐vWF immunohistochemistry staining revealed that AAV9‐miR‐486‐EGFP treatment was able to improve cardiac angiogenesis in the post‐MI heart. (H1) Representative images of the AAV9‐miR‐486‐EGFP‐ and AAV9‐NC‐treated groups in the infarct zone. (H2) Quantitative analysis of (H1). (H3) Representative images of the AAV9‐miR‐486‐EGFP‐ and AAV9‐NC‐treated groups in the border zone. **(**H4) Quantitative analysis of H3. *n* = 5 and 6 for the AAV9‐NC and AAV9‐miR‐486 groups, respectively.

Having identified the function and signalling pathways of miR‐486 in transdifferentiated CMFs in vitro, we next addressed the question of whether and how miR‐486 is involved in MI. First, we found that miR‐486 expression was downregulated significantly in the infarct zone and border zone 8 weeks after MI, accompanied by upregulation of *SRSF3* (Figure 5A, B), indicating the involvement of miR‐486 and SRSF3 in the pathophysiology of post‐MI myocardium.

To investigate its function, we first overexpressed miR‐486 via AAV9‐miR‐486‐EGFP intramyocardial injection in the infarct zone and border zone of MI hearts immediately after LAD ligation, leading to a significantly higher expression level of miR‐486 in the infarct zone than that of the AAV9‐NC‐treated group at 8 weeks post‐MI (Figure 5C; *p* < 0.01).

In parallel, in comparison with the control group, the AAV9‐miR‐486–EGFP‐treated group had a significantly thicker left ventricular anterior wall (LVAW) in the end‐systolic phase (Figure 5D‐D1,2) and end‐diastolic phase (Figure 5D‐D1,3), smaller left ventricular end‐systolic diameter (LVESD) (Figure 5D‐D4) but not left ventricular end‐diastolic diameter (LVEDD) (Figure 5D‐D5), and better ejection fraction (EF) (Figure 5D‐D6) and fractional shortening (FS) (Figure 5D‐D7) values by echocardiography, indicating less fibrotic pathology and cardiac remodelling and better cardiac function. Indeed, compared to the AAV9‐NC‐treated group, a smaller area of collagen in the infarct zone (CAIZ) (Figure 5E‐E2; *p* < 0.05), larger thickness of the infarcted myocardium of the left ventricle (TIM) (Figure 5E‐E3; *p* < 0.05) and wall thickness of the border zone of the left ventricle (WTBZ) (Figure 5E‐E4; *p* < 0.05), smaller infarct size (Figure 5E‐E5; *p* < 0.05), longer running endurance time by treadmill test (Figure 5F; *p* < 0.05) in the AAV9‐miR‐486–EGFP‐treated group also indicated less cardiac fibrotic area, fibrotic pathology and cardiac remodelling as well as a recovery in cardiac function. In addition, a smaller area of cardiomyocytes in both the border zone and remote zone by WGA staining (Figure 5G; *p* < 0.05) was identified in the AAV9‐miR‐486–EGFP‐treated group. Furthermore, anti‐vWF immunohistochemistry staining revealed that AAV9‐miR‐486‐EGFP treatment improved cardiac angiogenesis in post‐MI hearts (Figure 5H; *p* < 0.05). Importantly, the results of a 24‐week post‐MI analysis demonstrated that all the observed beneficial effects of AAV9‐miR‐486‐EGFP treatment in MI hearts were maintained for at least 24 weeks (Figure S2; *p* < 0.05). These in vivo results are in line with the gene expression in CMFs showing that miR‐486 treatment improves fibrotic pathology, fibrosis and pathological remodelling in post‐MI hearts, which benefits the regeneration of MI.

### 3.5 miR‐486 overexpression mediates cellular senescence of CMFs in vivo

**FIGURE 6:**
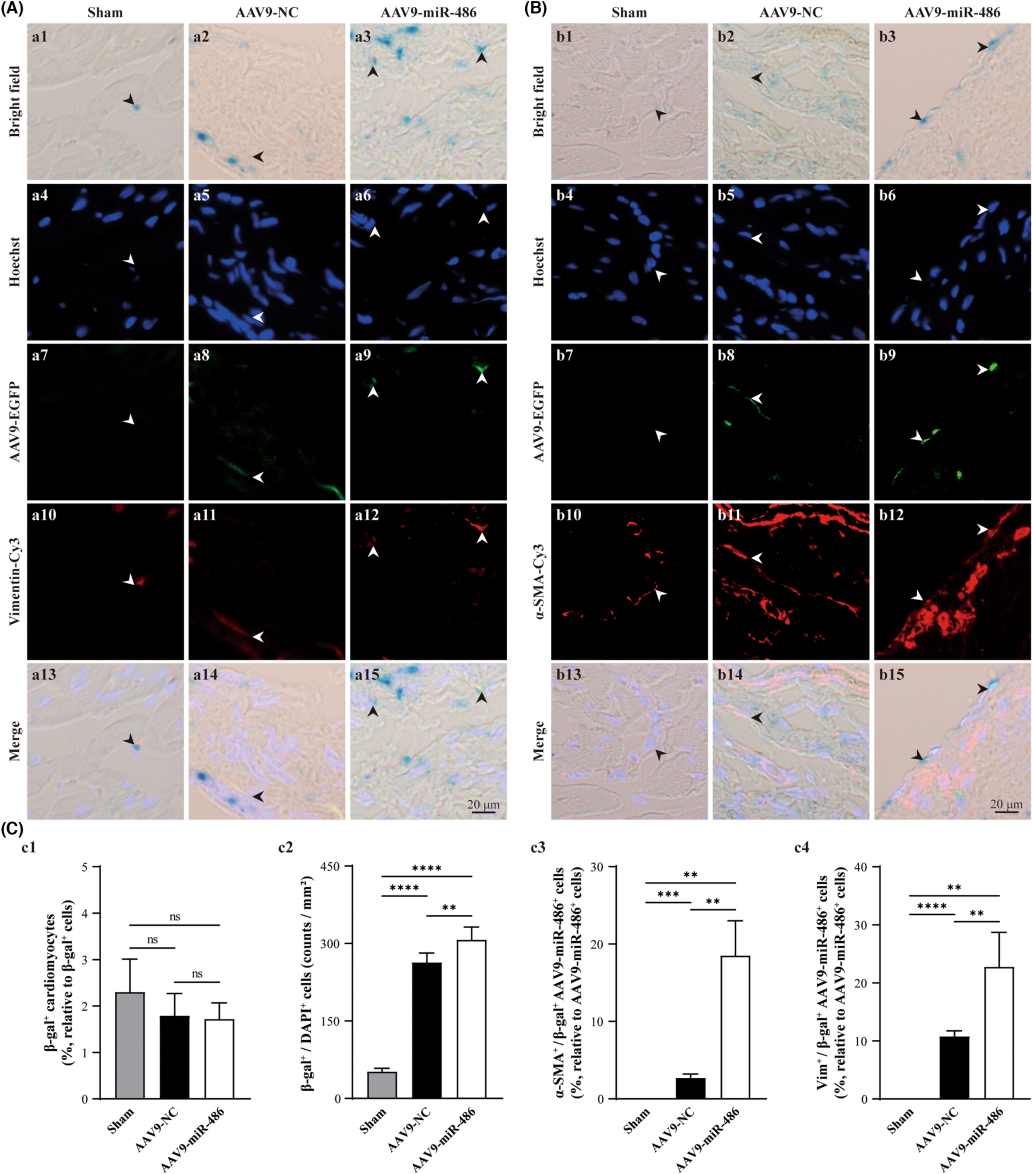
miR‐486 overexpression increases the cellular senescence of CMFs in the infarct zone but not cardiomyocytes in the post‐MI myocardium in vivo. (A) Representative images of β‐gal staining (blue under bright field), anti‐Vimentin (Cy3; red under fluorescence) staining and Hoechst staining (blue under fluorescence), which were conducted in the same AAV9‐miR‐486‐EGFP (green)‐transfected myocardium sections at 8 weeks post‐MI. (B) Representative images of β‐gal staining (blue under bright field), anti‐α‐SMA (Cy3; red under fluorescence) staining and Hoechst staining (blue under fluorescence), which were conducted in the same AAV9‐miR‐486‐EGFP (green)‐transfected myocardium sections at 8 weeks post‐MI. β‐gal‐positive staining was performed to label senescent cells. Vimentin was used as a marker for CFs and CMFs. Vimentin‐ and α‐SMA‐positive cells were used to identify CMFs. Hoechst was applied to identify the nucleus. (C‐C1) Semiquantification of β‐gal^+^/cTnI^+^ cardiomyocytes in cross sections of whole myocardium for senescent cardiomyocytes. (C‐C2) Semiquantification of β‐gal^+^/DAPI^+^ noncardiomyocytes in the infarct zone. (C‐C3) Semiquantification of α‐SMA^+^/β‐gal^+^/AAV9‐miR‐486‐EGFP^+^ cells vs. AAV9‐miR‐486‐EGFP^+^ cells (%) in the infarct zone to analyse miR‐486 overexpression‐induced senescent CMFs. (C‐C4) Semiquantitative analysis of Vimentin^+^/β‐gal^+^/AAV9‐miR‐486‐EGFP^+^ cells vs. AAV9‐miR‐486‐EGFP^+^ cells (%) in the infarct zone to analyse miR‐486 overexpression‐induced senescent CFs and CMFs. *n* = 3.

As observed in transdifferentiated CMFs in vitro, the improvement of scar formation and remodelling in post‐MI hearts through overexpression of miR‐486 could be attributed to the senescence state of CMFs, since CMFs are important players in scar formation post‐MI. To investigate the effect of miR‐486 in the infarct zone, we generated an AAV9 vector (AAV9‐miR‐486‐EGFP) containing miR‐486, which was linked with the EGFP‐encoding gene downstream of miR‐486. Green fluorescence was visualized to trace miR‐486‐expressing cells in vivo. In addition, using anti‐α‐SMA (marker for myofibroblasts), anti‐Vimentin (both positive in fibroblasts and myofibroblasts), anti‐CTnI (marker for mature cardiomyocytes) immunofluorescent staining and β‐gal staining (for cellular senescence), we were able to identify the cellular senescence of CMFs, CFs/CMFs and cardiomyocytes (CMs) in the same sections of AAV9‐miR‐486‐EGFP‐treated hearts (Figure 6A and B; Figure S3). Our results indicated that most of the senescent cells (β‐gal positive) were located in interstitial tissue in the AAV9‐miR‐486‐EGFP‐treated MI myocardium. Only a small number of CMs were found to be β‐gal positive. Moreover, miR‐486 treatment did not alter the percentage of β‐gal‐positive CMs to total β‐gal‐positive cells (Figure 6C‐C1 and Figure S3; *p* > 0.05).

In contrast, the density of β‐gal+hoescht‐positive cells in the AAV9‐miR‐486‐treated group was significantly higher than that in the AAV9‐NC‐treated group and sham group (Figure 6C‐C2, *p* < 0.05). Furthermore, miR‐486 treatment led to many more CFs and CMFs transforming to a senescent state. The percentage of α‐SMA^+^/β‐gal^+^/AAV9‐miR‐486‐EGFP^+^ CMFs to AAV9‐miR‐486‐EGFP^+^ cells (18.5%; miR‐486 overexpression‐induced senescence CMFs) in the AAV9‐miR‐486‐EGFP‐treated infarct zone was significantly higher than that in the AAV9‐NC‐treated infarct zone (2.7%) (Figure 6C‐C3; *p* < 0.05). In addition, in the AAV9‐miR‐486‐EGFP‐treated infarct zone, the percentage of Vimentin^+^/β‐gal^+^/AAV9‐miR‐486‐EGFP^+^ cells to AAV9‐miR‐486‐EGFP^+^ cells (22.76%; miR‐486 overexpression‐induced senescent CFs and CMFs) (Figure 6A‐A3) was approximately 4.26% higher than the percentage of α‐SMA^+^/β‐gal^+^/AAV9‐miR‐486‐EGFP^+^ CMFs (18.50%; miR‐486 overexpression‐induced senescent CMFs) (Figure 6C‐C3 vs. C4). The results showed that most of the miR‐486‐treated senescent cells were CMFs, while approximately 4.26% were senescent CFs. To further investigate whether miR‐486‐mediated senescent cells in vivo include inflammatory cells, double staining for anti‐CD68 (M1 macrophage marker; inflammatory macrophages), anti‐CD163 (M2 macrophage marker; anti‐inflammatory macrophages) and anti‐CD11b (neutrophil marker) combined with β‐gal staining was conducted. The results showed that CD68‐positive+β‐gal‐positive cells and CD11b‐positive+β‐gal‐positive cells were not found in the AAV9‐miR‐486‐treated infarct zone or AAV9‐NC‐treated infarct zone. In addition, the densities of CD68‐positive cells and CD11b‐positive cells in the AAV9‐miR‐486‐treated infarct zone were similar to those in the AAV9‐NC‐treated infarct zone (Figure S4A and B, *p* > 0.05). In addition, CD163‐positive cells were not found in the AAV9‐miR‐486‐treated infarct zone or AAV9‐NC‐treated infarct zone (Figure S4C, *p* > 0.05). Taken together, miR‐486 overexpression mediated the cellular senescence of CMFs (mainly) and CFs (only a small portion) but not cardiomyocytes or inflammatory cells (M1 and M2 macrophages and neutrophils). Thus, the senescence effect of miR‐486 on CMFs in post‐MI hearts is in accordance with the results from in vitro experiments with transdifferentiated CMFs.

### 3.6 miR‐486 decreases SRSF3 and increases *p*21 expression accompanied by a decrease in the expression of fibrosis effector genes in post‐MI hearts in vivo

**FIGURE 7:**
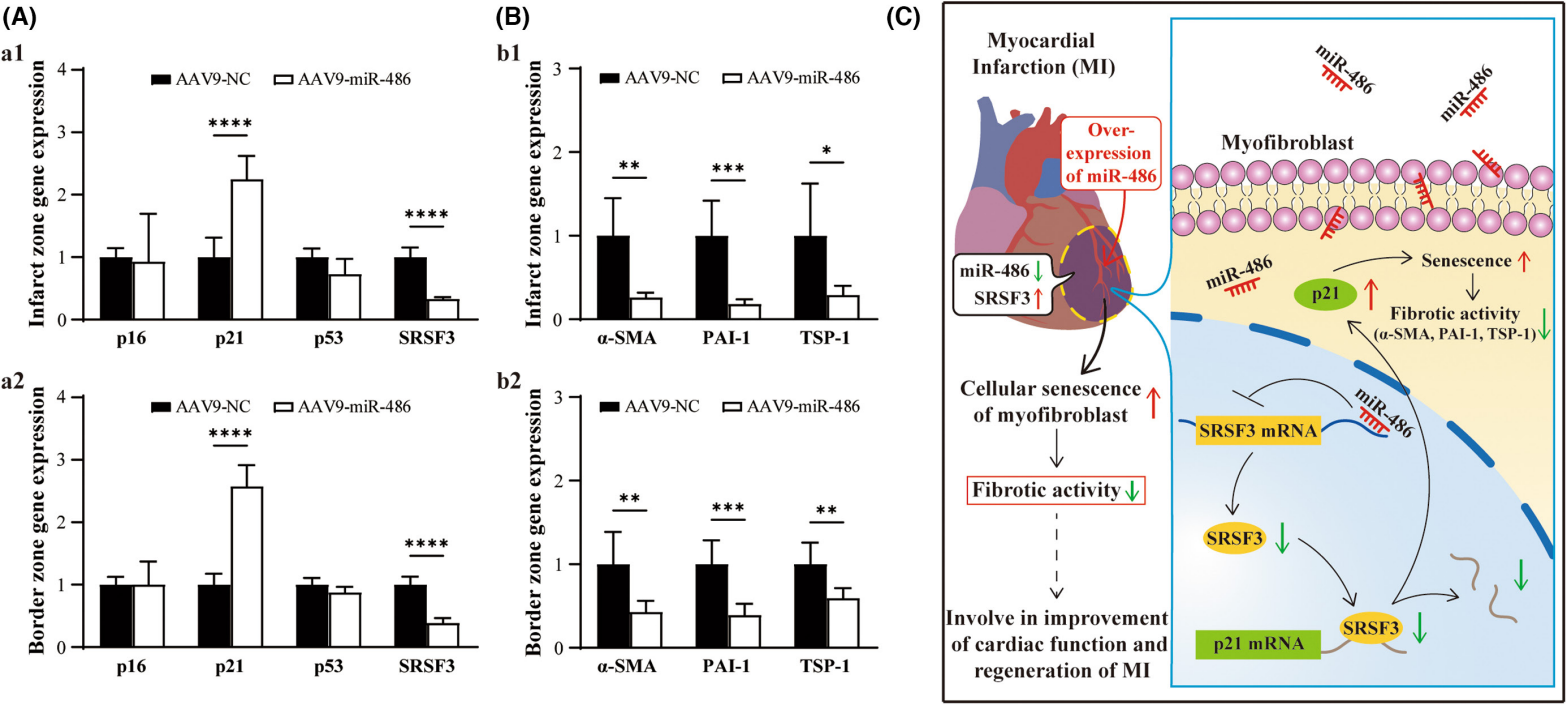
Overexpression of miR‐486 decreases SRSF3 expression, increases the expression of the senescence‐promoting gene *p21* and decreases the expression of fibrosis effector genes in post‐MI hearts in vivo. The below parameters were analysed 8 weeks post‐MI. (A) Overexpression of miR‐486 in MI myocardium increased *p21* expression and decreased *SRSF3* expression but not *p53* and *p16* expression in myocardial tissue in both the infarct zone and border zone. (B) Overexpression of miR‐486 in MI myocardium decreased the expression of fibrosis effector genes (*PAI‐1*, *TSP‐1 and α‐SMA*) in myocardial tissue in both the infarct zone and border zone. *n* = 3. (C) Schematic diagram of the molecular mechanism by which miR‐486/SRSF3/*p*21 targets senescence in CMFs to improve MI regeneration. In the infarct zone of MI, the expression of miR‐486 was downregulated, but the expression of SRSF3 was upregulated. miR‐486 targets silencing of SRSF3, and upregulation of *p*21 mediates the senescence of cardiac myofibroblasts to improve their fibrotic activity and limit scar size and post‐MI remodelling, which benefits the regeneration of MI

To investigate whether the miR‐486/SRSF3/*p*21 pathway, which was identified in an in vitro experiment with transdifferentiated CMFs, functions in post‐MI hearts in vivo, miR‐486 was overexpressed in MI as described above. 8 weeks after AAV9‐miR‐486‐EGFP treatment in MI hearts in vivo, overexpression of miR‐486 in MI myocardium decreased *SRSF3* and increased *p21* expression in myocardial tissue in both the infarct zone and border zone, while *p53* and *p16* expression were not significantly altered, as observed in vitro (Figure 7A; *p* < 0.05). In addition, the expression of fibrosis effector genes (*PAI‐1*, *TSP‐1 and α‐SMA*) in AAV9‐miR‐486‐EGFP‐treated infarct and noninfarct tissues was significantly decreased compared with that in AAV9‐NC‐treated infarct and noninfarct tissues (Figure 7B, C; *p* < 0.05). The results together with the above in vivo findings suggest that miR‐486 improves fibrotic activity in myocardial infarction by targeting the SRSF3/*p*21 pathway to mediate cardiac myofibroblast senescence in vivo.

## 4 DISCUSSION

In the present study, our in vitro studies demonstrated that overexpression of miR‐486 in CMFs induced cellular senescence and apoptosis and decreased proliferation and cell cycle inhibition at the S and G2/M phases. In addition, miR‐486 overexpression was able to induce more senescent CMFs than apoptotic CMFs. Most miR‐486‐treated senescent CMFs did not undergo apoptosis simultaneously, which is consistent with the well‐established knowledge that senescent cells are usually resistant to apoptosis.^30^, ^31^ Furthermore, overexpression of miR‐486 mediated activation of the *p*21 cellular senescence pathway and decreased the expression of well‐established fibrosis effector genes (*PAI‐1*, *TSP‐1 and α‐SMA*) in CMFs. Importantly, the downstream functional target gene of miR‐486, which is involved in the upregulation of the *p21* gene and downregulation of fibrosis effector genes (*PAI‐1*, *TSP‐1 and α‐SMA*) in CMFs, was identified as *SRSF3*. In parallel, our in vivo study further confirmed that downregulation of miR‐486 accompanied by upregulation of *SRSF3* occurred in the infarct zone post‐MI, which suggested that miR‐486 and SRSF3 were involved in the pathophysiology of MI. Indeed, our in vivo study revealed that miR‐486 overexpression in MI increased the density of senescent CMFs but not the density of senescent cardiomyocytes and inflammatory cells (M1 and M2 macrophages and neutrophils), and the expression of the cellular senescence‐promoting gene *p21* was accompanied by downregulation of the expression of *SRSF3* and fibrosis effector genes (*PAI‐1*, *TSP‐1* and *α‐SMA*) in both the infarct zone and border zone. Furthermore, it was found that miR‐486 overexpression in MI led to a decrease in infarction area, improved cardiac function and pathological remodelling and promoted cardiac angiogenesis. Thus, in vitro findings combined with the in vivo results in the present study uncovered a novel mechanism by which miR‐486 improves fibrotic activity, pathologic remodelling and scar size in MI via an *SRSF3* gene silencing‐mediated increase in *p*21 signalling resulting in cellular senescence of CMFs, which benefits MI regeneration. Indeed, recent studies have documented that miR‐486‐5*p* plays a crucial role in the suppression of hypertropic scar fibroblasts^16^ and the regulation of cellular senescence in human diploid fibroblasts^17^ and is involved in IgE elevation‐mediated pathological cardiac fibrosis.^18^ Our findings and those of others clearly indicate that miR‐486 is a critical endogenous player that balances fibrotic activity in fibrotic‐producing cells and myofibroblasts. In addition, our findings showing that miR‐486 expression is downregulated and *SRSF3* expression is upregulated in ischaemic myocardium, especially in the infarct zone, combined with the above novel mechanism, strongly suggest that the miR‐486/*SRSF3/p21* pathway, which mediates senescence of CMFs, is a potential therapeutic target to improve the fibrotic activity and pathological fibrosis and remodelling seen in ischaemic myocardium, such as MI, and to benefit regeneration.

In fact, recent studies have revealed that under hypoxic and ischaemic situations, miR‐486‐5*p* found in bone marrow stromal cell‐derived exosomes inhibits cardiomyocyte apoptosis by targeting the PTEN/PI3K/AKT signalling pathway.^29^ miR‐486‐5*p* targeting *PTEN* to activate PI3K/AKT signalling protected against cardiomyocyte apoptosis mediated by coronary microembolization.^32^ In addition, studies of the in vitro rat embryonic ventricular cardiomyocyte cell line H9c2, revealed that miR‐486 targeting the silencing of *NDRG2* to inactivate JNK/C‐Jun and NF‐κB signalling improves hypoxia‐induced damage to cardiomyocytes.^33^ More recently, miR‐486‐5*p* found in stem cell‐secreted microvesicles was reported to increase angiogenesis and regeneration of MI via fibroblast MMP19‐VEGFA cleavage signalling in mice and nonhuman primates.^34^ In this study, we also verified that overexpression of miR‐486 in MI was able to promote cardiac angiogenesis in both the infarct zone and border zone. Thus, findings from our group and others suggest that in addition to targeting *SRSF3/p21* to mediate the senescence of CMFs, miR‐486 also has the potential to target cardiomyocytes and endothelial cells in the myocardium via multiple pathways to exert beneficial effects on the survival of cardiomyocytes and cardiac angiogenesis in ischaemic myocardium and facilitate the regeneration of MI. Importantly, the findings of the present study further revealed that miR‐486 treatment failed to induce cellular senescence of cardiomyocytes and inflammatory cells (M1 and M2 macrophages and neutrophils) in vivo. Therefore, our findings further reveal clearly that miR‐486 mediates the cellular senescence of CMFs, but not cardiomyocytes or inflammatory cells (M1 and M2 macrophages and neutrophils), to improve fibrotic activity and pathological fibrosis and remodelling, which is involved in improvement of MI regeneration.

Recent progress has demonstrated that cellular senescence is an important physiopathological process, and it has been documented to play different roles in various diseases, such as fibrotic pulmonary disease and liver fibrosis.^35^, ^36^ One of the important advancements in the study of cellular senescence is that triggering cellular senescence plays a critical role in promoting regeneration and limiting fibrosis in tissues and organs.^8^, ^9^, ^37^, ^38^, ^39^, ^40^ It has been demonstrated that in cutaneous wound healing, CCN1 is dynamically expressed at sites of inflammation and wound healing.^41^ CCN1 is able to induce cellular senescence of fibroblasts by targeting integrin α6β1 and heparan sulphate proteoglycans, which induce activation of the p16INK4a/pRb senescence pathway.^38^ In addition, Trp53‐mediated cardiac fibroblast senescence limits cardiac fibrosis in adult MI hearts.^7^ Apical resection‐induced cardiomyocyte CCN1 secretion results in fibroblast senescence, which enhances the proliferation of cardiomyocytes and decreases cardiac fibrosis to improve neonatal heart regeneration.^9^ Furthermore, the cellular senescence of CMFs was identified as an essential antifibrotic mechanism in the adult myocardium.^8^ In contrast to current knowledge, the present study proposes a novel regulatory mechanism by which miR‐486 induces cellular senescence in CMFs via an *SRSF3* gene silencing‐mediated increase in p21 signalling. The identified mechanism, together with the finding that miR‐486‐mediated *SRSF3* silencing simultaneously decreases the expression of fibrosis effector genes (*PAI‐1*, *TSP‐1* and *α‐SMA*), which are well‐established important activators of fibrotic activity,^3^, ^22^, ^23^, ^24^, ^25^, ^26^ improves cardiac fibrosis and post‐MI remodelling, thereby providing beneficial effects to enhance MI regeneration. Indeed, the findings of this study also demonstrated that miR‐486 treatment promotes cardiac angiogenesis, decreases infarct size and improves cardiac function in MI.

The results of the present study also showed that the expression of *p*53 and *p*16 was upregulated in miR‐486‐treated CMFs but not in si*SRSF3*‐treated CMFs. This result indicated that the therapeutic effects of miR‐486 treatment in MI, such as promoting cardiac angiogenesis and increasing the expression of *p*53 and *p*16, cannot be attributed to *SRSF3* as the target gene. In fact, the existence of multiple target genes is a well‐identified mechanism for miRNA‐mediated gene silencing.^42^ Indeed, miR‐486 was reported to connect myostatin and the IGF‐1/Akt/mTOR pathway to regulate the size of skeletal muscle^43^ and target TGF‐β2‐Smad2/3 signalling‐mediated suppression of epithelial cell proliferation.^44^ In addition, miR‐486‐5*p* was found to target IGF1/PI3K/AKT signalling to limit the proliferation and collagen production of hypertrophic scar fibroblasts.^16^ The mechanism and pathway underlying miR‐486‐mediated cardiac angiogenesis and increased expression of *p*53 and *p16* in the present study need to be studied in‐depth in future investigations.

Serine/arginine‐rich proteins (SR proteins) belong to the RNA binding protein family, which plays an important role in pre‐mRNA constitutive and alternative splicing. Among twelve SRSFs (named SRSF1‐SRSF12) that have been identified in humans, SRSF3 is the smallest.^45^ The SRSF3 protein (64 amino acids and approximately 19 kDa) consists of two domains, namely an RNA recognition domain (RRM; N‐terminal domain) and an arginine/serine domain (RS; C‐terminal domain).^45^ The RS domain acts to target SRSF3 to nuclear speckles.^46^ SRSR3 identifies the CUC(U/G)UCY splicing enhancer sequence to conduct RNA alternative splicing.^47^ SRSF3 was reported to be involved in alternative splicing of *p53* mRNA towards the *p*53β isoform to induce cellular senescence,^27^ heart development and MI.^48^ Recently, it was identified that decreased SRSF3 expression results in preferential usage of the proximal poly(A) site of mRNA (shortening the 3'UTR) in human and mouse cells to produce more protein, which induces cellular senescence by increasing the production of senescence‐associated genes (*PTEN*, *PIAS1* and *DNMT3A*).^28^ Here, we further revealed that SRSF3 is a novel target gene of miR‐486. miR‐486 is able to mediate the targeted silencing of *SRSF3* accompanied by upregulation of *p21* in CMFs in vitro and in the infarct zone and border zone of MI. Furthermore, RIP‐qPCR results showed that the SRSF3 protein is able to bind with *p21*. In view of the well‐established functions of SRSF3 in alternative splicing and knockdown of *SRSF3* expression resulting in preferential usage of shortened 3'UTRs of mRNAs that produce more protein, our findings suggested that SRSF3 might utilize a similar mechanism to trigger cellular senescence via *p21* upregulation when miR‐486‐targeted silencing of *SRSF3* occurs. The exact molecular mechanism by which SRSF3 induces *p*21 upregulation‐mediated cellular senescence needs to be investigated in‐depth in future studies. In addition, it is well established that in the *p*53/*p*21‐mediated cellular senescence pathway, *p*53 is an upstream effector for the upregulation of *p*21.^49^, ^50^ However, even though our in vitro study showed that miR‐486 overexpression in CMFs was able to upregulate *p*21, *p*53 and *p*16 expression, the results of the siSRSF3 in vitro treatment for CMFs and in vivo study did not show the effect of *p*53 and *p*16 upregulation upon overexpression of miR‐486 in the myocardium. This suggests that miR‐486‐targeted silencing of SRSF3 and *p*21 upregulation mediates the senescence of CMFs, which might be due to direct binding of *p*21 but not to activation of *p*53 as its upstream signalling molecule.

In summary, a novel molecular mechanism was uncovered in the current study, in which miR‐486 improved fibrotic activity, pathological remodelling and scar size via an *SRSF3* gene silencing‐mediated increase in *p*21 signalling to induce cellular senescence of CMFs, resulting in beneficial effects for the improvement of MI regeneration. Therefore, the pathway by which miR‐486 targets *SRSF3/p21* to mediate the senescence of CMFs is a potential therapeutic target to improve the fibrotic activity and pathological fibrosis and remodelling seen in ischaemic myocardium, such as MI, to promote increased regeneration.

## AUTHOR CONTRIBUTIONS

**Hongyi Chen:** Conceptualization (supporting); data curation (lead); formal analysis (lead); investigation (lead); methodology (lead); software (lead); writing – original draft (supporting). **Luocheng Lv:** Methodology (supporting). **Ruoxu Liang:** Methodology (supporting). **Weimin Guo:** Methodology (supporting). **Zhaofu Liao:** Methodology (supporting). **Yilin Chen:** Methodology (supporting). **Kuikui Zhu:** Methodology (supporting). **Ruijin Huang:** Conceptualization (supporting). **Hui Zhao:** Conceptualization (supporting). **Qin Pu:** Conceptualization (supporting). **Ziqiang Yuan:** Conceptualization (supporting). **Zhaohua Zeng:** Conceptualization (supporting). **Xin Zheng:** Data curation (supporting). **Shanshan Feng:** Conceptualization (supporting). **Xu‐Feng Qi:** Conceptualization (supporting). **Dongqing Cai:** Conceptualization (lead); funding acquisition (lead); project administration (lead); resources (lead); supervision (lead); writing – original draft (lead); writing – review and editing (lead).

## CONFLICT OF INTEREST

The authors declare no conflict of interest.

## Supporting information


**Figure** **S1**

**Figure** **S2**

**Figure** **S3**

**Figure** **S4**

**S1** Supporting information

## Data Availability

The data that support the findings of this study are available from the corresponding author upon reasonable request.
