# Supplementary figures and images for: miR‐486 improves fibrotic activity in myocardial infarction by targeting SRSF3/p21‐Mediated cardiac myofibroblast senescence
Source: J Cell Mol Med. 2022 Sep 18;26(20):5135–49. doi: 10.1111/jcmm.17539 (PMC9575141; doi:10.1111/jcmm.17539)

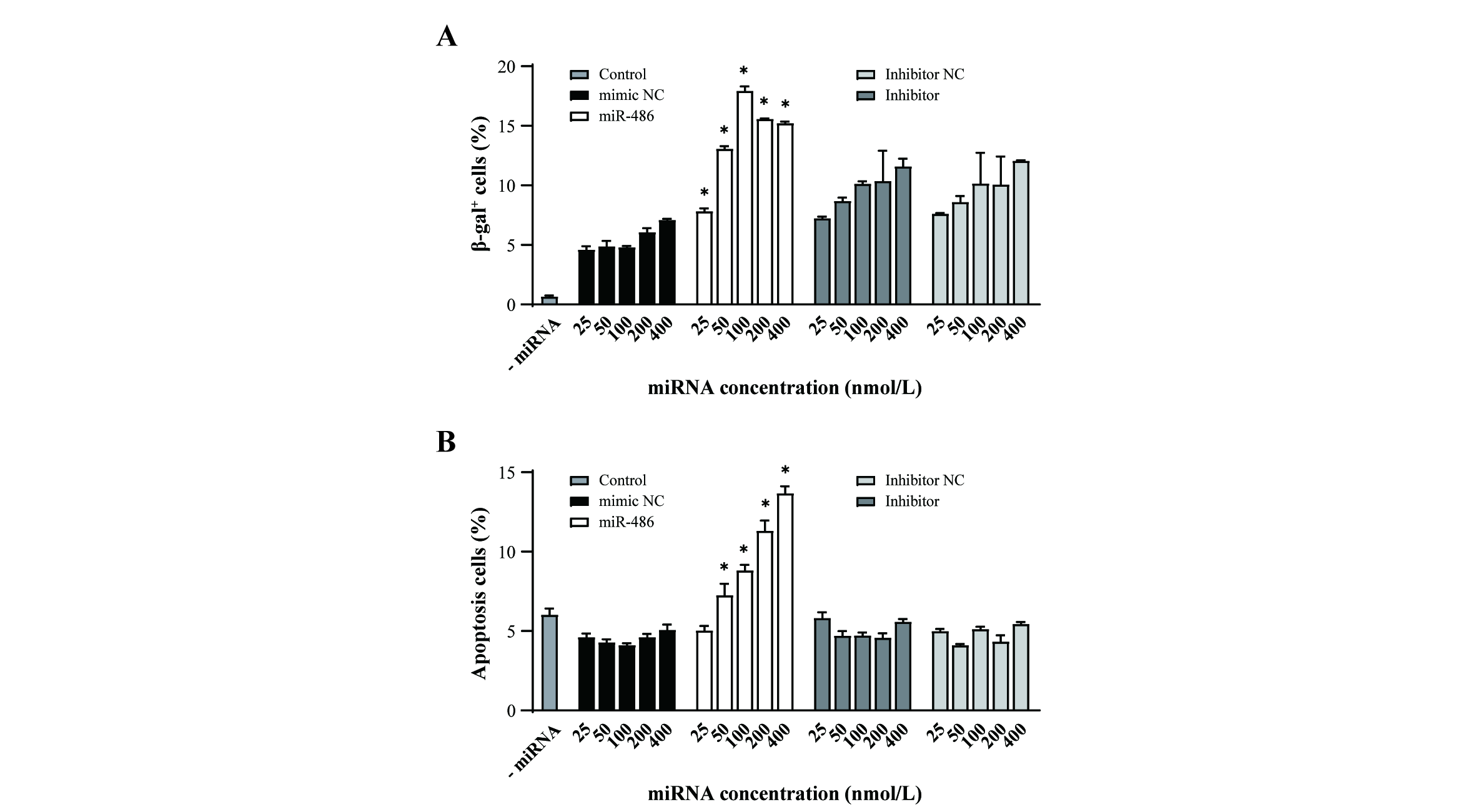

Supplement: Supplementary file 1 — Figure S1 [file JCMM-26-5135-s003.tif]

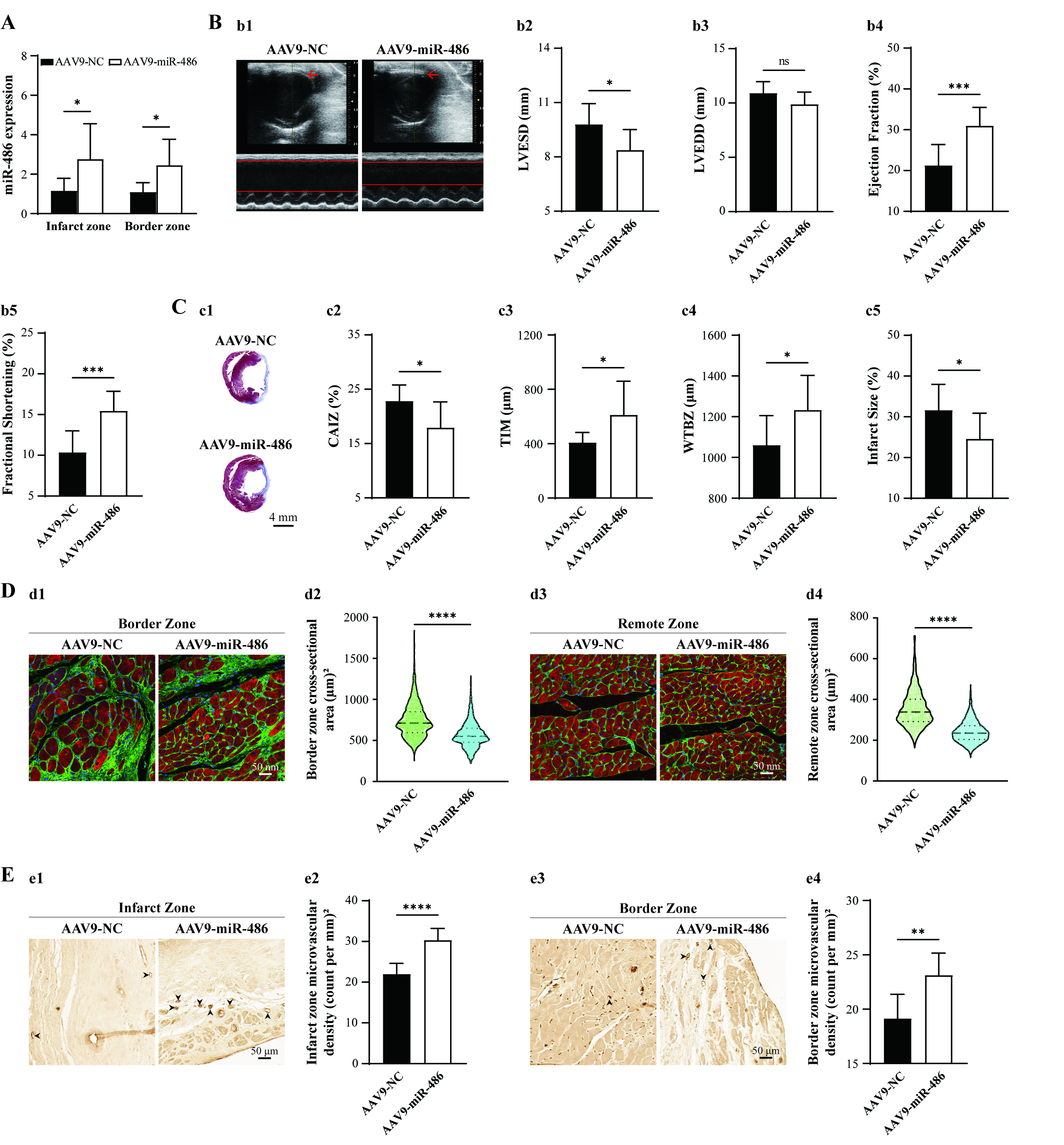

Supplement: Supplementary file 2 — Figure S2 [file JCMM-26-5135-s002.tif]

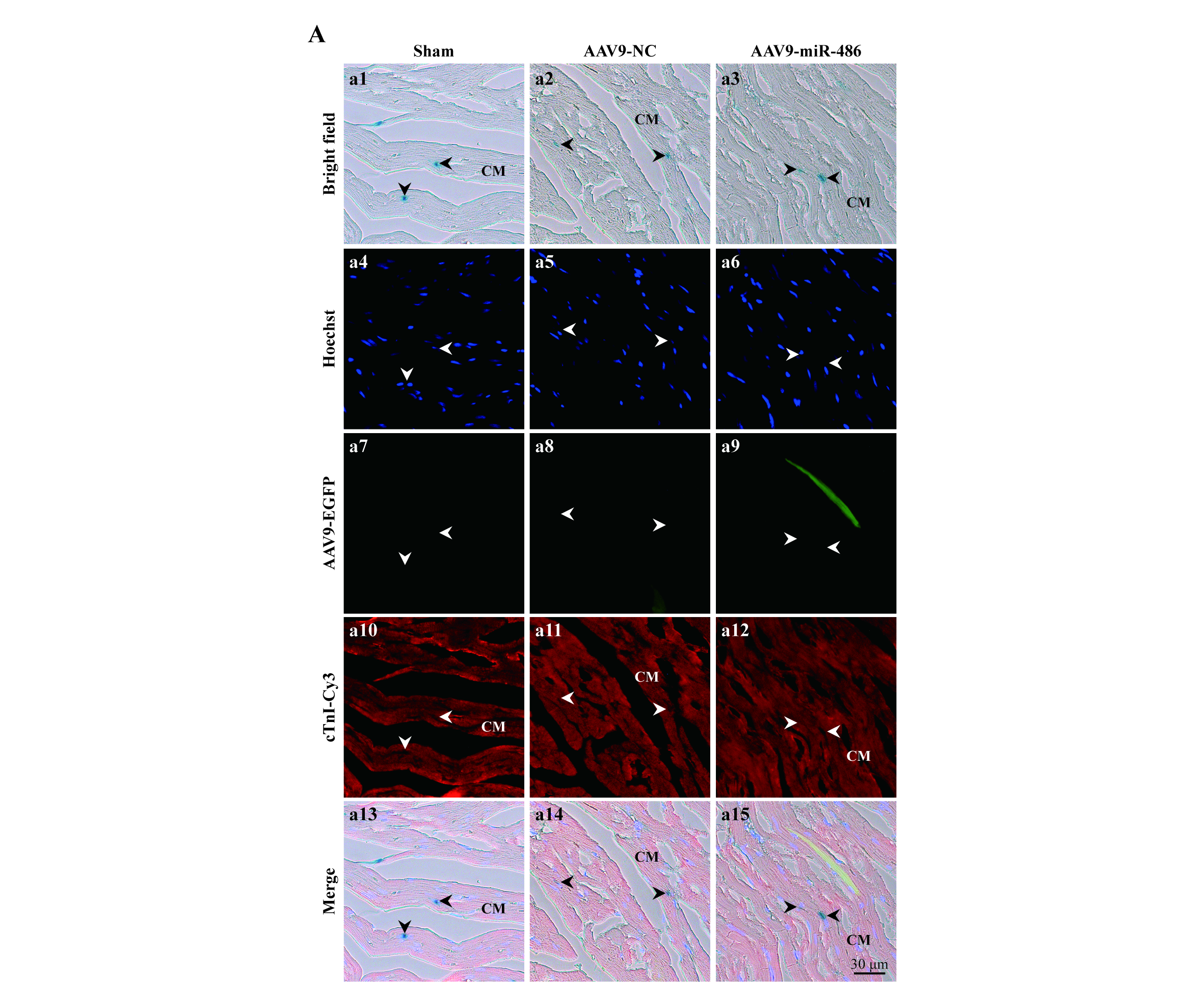

Supplement: Supplementary file 3 — Figure S3 [file JCMM-26-5135-s005.tif]

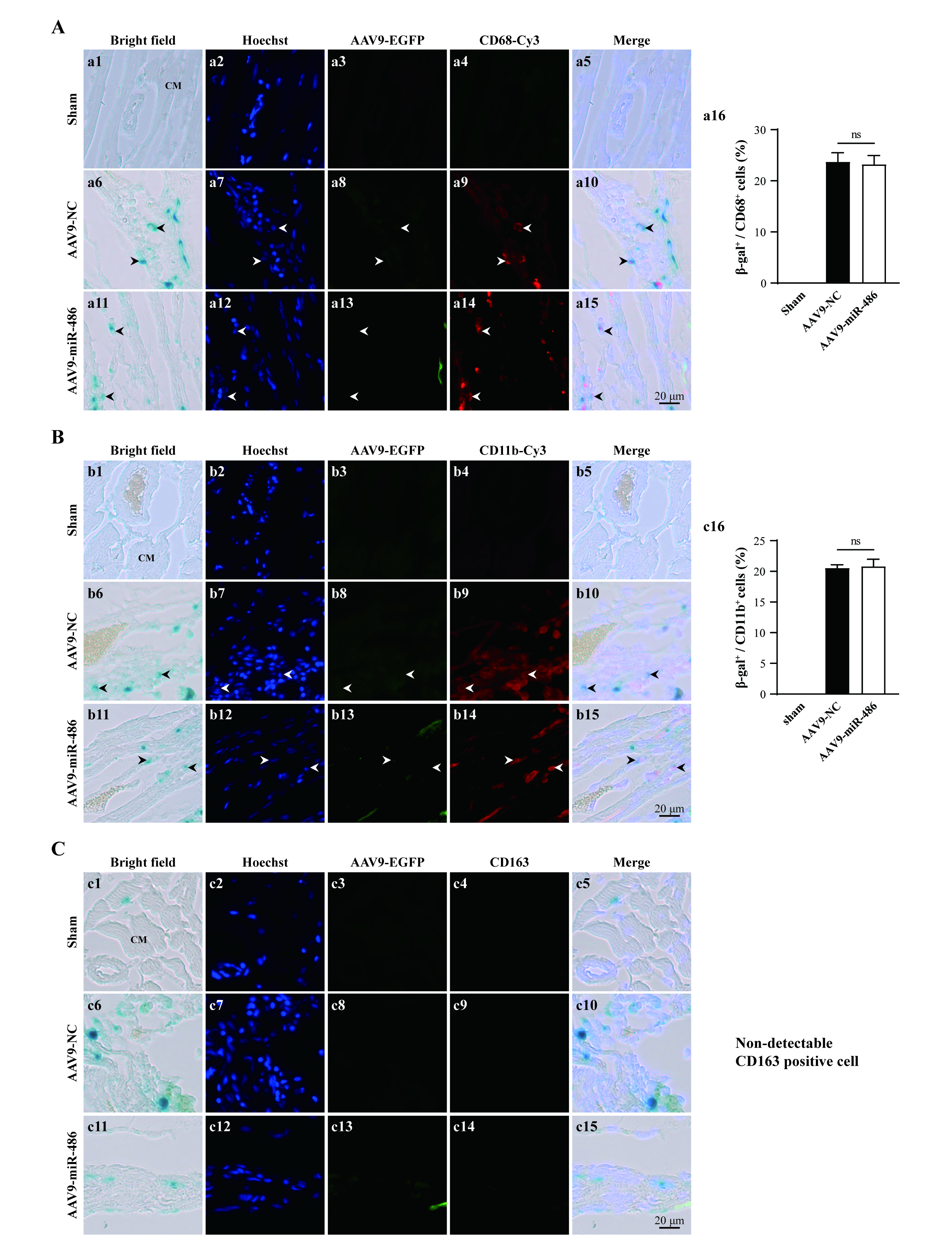

Supplement: Supplementary file 4 — Figure S4 [file JCMM-26-5135-s004.tif]
