# Supplementary material for: miR‐486 improves fibrotic activity in myocardial infarction by targeting SRSF3/p21‐Mediated cardiac myofibroblast senescence
Source: J Cell Mol Med. 2022 Sep 18;26(20):5135–49. doi: 10.1111/jcmm.17539 (PMC9575141; doi:10.1111/jcmm.17539)
Supplement: Supplementary file 5 — S1 Supporting information [file JCMM-26-5135-s001.docx]

**Appendix**

**Animals**

In present study, two-month-old female Sprague–Dawley (SD) rats (200-250 g) were used. The rats were adapted for 1 week feeding before experimentation. The animals were provided with food and water *ad libitum*. Animal care, surgery and handling procedures were approved by the Jinan University Animal Care Committee (Approval No. IACUC-20190104-010).

**Preparation and culture of CMFs**

CFs were isolated from the ventricles of 2-month-old female SD rats as published reported ^19^. Briefly, the ventricles were minced and treated with DMEM supplemented with 0.05% collagenase P and 0.1% trypsin and incubated for 30 min at 37 ℃ on a shaker (180 rpm). The digested tissue was dissociated by pipetting every 10 min. The supernatant was then filtered through a 41-μm nylon mesh, and the collected cell suspension was centrifuged at 50 × g for 2 min. The supernatant was then removed and recentrifuged at 1600 rpm for 4 min to collect cells. The pellet was suspended in growth medium and plated onto a 60 mm cell culture dish. The growth medium was DMEM supplemented with 10% FBS and 1% penicillin/streptomycin at 37 ℃ and 5% CO_2_ in a 95% air incubator. Following 1 h of incubation, the medium was changed, with the adherent CFs remaining on the plate. The marker of CFs, Vimentin, was applied to confirm the correct phenotype of CFs. For the preparation of CMFs, 10 ng/mL TGF-β1 (cat. no. 5231LC; CST) was added to cultured CFs to induce CMF differentiation for 48 hours. The gene expression of *α-SMA* and *Col1a1* combined with anti-α-SMA immunofluorescence was applied to confirm successful CMF transdifferentiation. In the present study, only prepared CMFs at passage 5 or lower were used for experiments. The studies were conducted using three animals in at least three repeated experiments for each individual animal.

**Immunofluorescence of Vimentin, α-SMA, cTnI, CD68, CD163 and CD11b**

The prepared cells or frozen sections were fixed with 4% paraformaldehyde for 15 min, after blocked with 1% BSA at room temperature for 1 h, then incubated with anti-Vimentin (1:200; cat. no. ab8978; Abcam), anti-α-SMA (1:200; cat. no. ab5694; Abcam), anti-cTnI (1:100; cat. no. ab47003; Abcam), anti-CD68 (1:100; cat. no. MCA341R; AbD), anti-CD163 (1:200; cat. no. ab182422; Abcam) and anti-CD11b (1:200; cat. no. ab133357; Abcam) at 4 ℃ overnight. After three-washing with PBS (pH=7.4), sequentially Alexa Fluor 488-conjugated secondary antibodies or Alexa Fluor 555-conjugated secondary antibodies were added respectively for 1 h. Finally, stained with Hoechst 33342 for 15 min after three-washing. The stained cells or sections were sealed with Antifade Mounting Medium (P0126; Beyotime). The cells or sections were observed under a microscope. The study was conducted using three identical wells in at least three repeated experiments.

**Isolation of total RNA and real-time quantitative PCR**

TRIzol reagent (cat. no. 15596018; Invitrogen) was used to extract Total RNA. The Hairpin-it miRNA q-PCR Quantitation Kit (cat. no. E01006-E01015; GenePharma) was applied to quantify rno-miR-486 expression. The specific stem-loop primers of rno-miR-486 (GenePharma) were used to reverse transcript the RNA (1μg). The expression level of miR-486 was analyzed by SYBR Green-based real-time PCR. The reaction mixture was composed of 10 μL of 2 × SYBR Green PCR Master Mix (cat. no. B21202; Biotool), 0.32 μL of miR-486 primers, 7.68 μL of nuclease-free water and 2 μL of the cDNA template. Amplifications were performed on a Mini-Opticon System (Bio-Rad) using the following program: initial denaturation at 95 ℃ for 3 min followed by 40 cycles of amplification at 95 ℃ for 12 s and then 62 ℃ for 40 s. Ct values were averaged and normalized to U6. Relative expression was determined using the 2^-ΔΔCt^ comparative threshold method. The study was conducted using three identical wells in at least three repeated experiments.

For analysis of the expression of the genes as Table-S1, ReverTra Ace q-PCR RT Master Mix with gDNA Remover (cat. no. FSQ-301, Toyobo) was used to reverse transcript the RNA (1μg), and the gene expression level was analyzed by SYBR Green-based real-time PCR. The reaction mixture was composed of 10 μL of 2 × SYBR Green PCR Master Mix, 1 μL of each primer, 6 μL of nuclease-free water and 2 μL of the cDNA template. Amplifications were performed on a Mini-Opticon System (Bio-Rad) using the program: initial denaturation at 95 ℃ for 3 min followed by 40 cycles of amplification at 95 ℃ for 15 s and then 62 ℃ for 30 s. Ct values were averaged and normalized to GAPDH.

**Transfection of rno-miR-486 mimics in CMFs**

The isolated CMFs were cultured to approximately 80% confluency with complete culture medium at 37 ℃ and 5% CO_2_ in a 95% air incubator and then transfected with rno-miR-486 mimics (5'-UCCUGUACUGAGCUGCCCCGAG-3'; 100 nM; GenePharma), the mimic negative control (5'-UUGUACUACACAAAAGUACUG-3'; 100 nM; GenePharma), the miR-486 inhibitor (5'-CUCGGGGCAGCUCAGUACAGGA-3'; 100 nM; GenePharma), and the inhibitor negative control (5'- UUGUACUACACAAAAGUACUG-3'; 100 nM; GenePharma) respectively using Lipofectamine RNAiMAX Kit (cat. no. 13778150; Invitrogen). The transfected cells were applied for qPCR assay, cell proliferation assay, cell apoptosis assay, cell cycle analysis, β-gal staining and flow cytometry analysis, Western blot assay and dual luciferase reporter assay. In present study, for the above assays (except dual luciferase reporter assay), 3 identical wells were observed in each analysis, and three repeat experiments were conducted. The study was conducted using three identical wells in at least three repeated experiments.

**β-galactosidase staining**

The prepared cells or frozen sections were used for senescence β-gal staining using a β-galactosidase staining kit (C0602; Beyotime) according to the manufacturer’s protocol. For staining in sections, β-gal-positive cells were counted in the infarct zone and border zone in the MI group (treated and nontreated control groups), while β-gal-positive cells in the corresponding area in the left ventricle were counted in the sham group. The numbers of β-gal-positive cardiomyocytes (CMs) were counted by combining the unique morphology of sarcomeres and cross-striatal structures. The constructed AVV9-derived vector (AVV9-miR-486-EGFP) in which overexpressed miR-486 was linked to the EGFP-encoding gene (green fluorescence) downstream of miR-486 was used to monitor the expressed miR-486 in transfected cells *in vivo*. Accordingly, by evaluating the green fluorescence positive combined with anti-α-SMA (marker for myofibroblasts) or anti-Vimentin (both positive in fibroblasts and myofibroblast) immunofluorescent staining (red fluorescence), β-gal staining (for cellular senescence) and unique morphology of cardiomyocyte in same section, we were able to analyze the cellular senescence of CMFs and CMs in the AVV9-miR-486-EGFP treated heart sections. The study was conducted using three identical wells in at least three repeated experiments.

**β-galactosidase fluorescence assay**

After prepared CMFs were treated with miR-486 for 48 hours, cellular senescence was analyzed using the ImaGene Green^TM^ C_12_FDG lacZ Gene Expression Kit (I-2904; Invitrogen) according to the manufacturer’s protocol. The senescent cells were quantitated by flow cytometry. The mimic negative control, inhibitor and inhibitor negative control groups were analyzed as controls. The study was conducted using three identical wells in at least three repeated experiments.

**Cell proliferation assay**

A CCK-8 assay (CK04; Dojindo) was performed to analyze the proliferation of CMFs after miR-486 treatment for 48, 72 and 96 hours. The detailed procedure followed the manufacturer’s protocol. Briefly, CCK-8 mixed medium was added and cultured for 1 hour. The absorbance value (at 450 nm) was analyzed. The mimic negative control, inhibitor and inhibitor negative control groups were tested as controls. The study was conducted using three identical wells in at least three repeated experiments.

**Apoptosis assay**

CMFs were cultured at 37 ℃ and 5% CO_2_ in a 95% air incubator. The Annexin Alexa Fluor 488/PI kit (FXP022-100, 4A Biotech) and subsequent flow cytometry analysis were applied to investigate the apoptosis of miR-486 treated CMFs according to the manufacturer’s instructions. Briefly, the treated CMFs (5×10^5^) were washed with PBS, resuspended in 100 μL of binding buffer and stained with 5 μL of Alexa Fluor 488-conjugated Annexin V. Following a 5 min incubation in a dark room, 10 μL of PI and 400 μL of PBS were added, and then the cells were analyzed using a flow cytometer (Cytoflex; Beckman Coulter). A total of 10,000 events were collected and analyzed for each sample. The mimic negative control, inhibitor and inhibitor negative control groups were tested as controls. The study was conducted using three identical wells in at least three repeated experiments.

**Cell cycle assay**

PI/RNase Staining Buffer (550825; BD) was applied to analyze the cell cycle of CMFs after miR-486 treatment for 72 hours. The collected cells were washed with PBS (pH=7.4) and centrifuged at 1600 rpm for 4 min. After the supernatant was removed, cold 70% ethanol was added, mixed with the cell pellet, and then incubated at -20 ℃ overnight. The mixture was centrifuged at 1600 rpm for 4 min to remove the supernatant, and the cell pellet was washed with PBS and again centrifuged at 1600 rpm for 4 min to remove the supernatant. The stained cells were resuspended in 0.5 mL of PI/RNase staining buffer, incubated at room temperature for 15 minutes, and then analyzed by flow cytometry. ModiFit Software was used for cell cycle analysis. The mimic negative control, inhibitor and inhibitor negative control groups were also observed as controls. The study was conducted using three identical wells in at least three repeated experiments.

**Western blot analysis**

The radioimmunoprecipitation assay (RIPA) buffer (cat. no. P0013C; Beyotime) containing a protease inhibitor cocktail (cat. no. W2200s; Cwbio) was applied to prepare the CMFs lysates. The total protein concentration was analyzed using a BCA Protein Assay Kit (cat. no. P0010; Beyotime). The extracted total protein of CMFs (25 μg) was denatured in loading buffer (5×SDS-PAGE loading buffer: 0.5 M Tris-HCl, pH 6.8, 0.05% beta-mercaptoethanol, 1% SDS, 0.005% bromophenol blue, 50% glycerol) for 10 min at 95 ℃, electrophoresed on a 12% SDS-PAGE gel, and then transferred to a PVDF membrane (cat. no. 162-0177; Bio-Rad). After blocked with 5% nonfat milk in TBST buffer, the PVDF membranes were incubated with the appropriate primary antibodies, including anti-CDKN2A/p16^INK4a^ (1:1000; cat. no. ab189034; Abcam), anti-p21 (1:1000; cat. no. ab109199; Abcam), anti-p53 (1:1000; cat. no. ab131442; Abcam), anti-SRSF3 (1:1000; cat. no. H00006428-M08; Novus), and anti-GAPDH (1:20000; cat. no. 60004-1-Ig; Proteintech) for overnight respectively. After washed with TBST, the prepared PVDF membranes were incubated with horseradish peroxidase (HRP)-conjugated secondary antibodies. The protein bands were visualized and imaged using an enhanced chemiluminescence system on a chemiluminescence reader (GeneGnome HR; Synoptics). The study was conducted using three identical wells in at least three repeated experiments. Please note that the Western blot band of GAPDH in Figure 2B-b1 and Figure 3C-c1, serving as the internal control, is the same image, as the two figures were obtained from the same piece of gel for electrophoresis separation, membrane transfer, and subsequent analysis. The same experimental setting for GAPDH was also for Figure 4B-b1 and Figure 4E-e1.

**Prediction of miR-486 targeted genes**

TargetScan (http://www.targetscan.org) was used to predict the potential target genes of rno-miR-486. FirePlex Discovery Engine (https://www.fireflybio.com/ portal/search) was used to verify whether or not a gene is already reported as target gene for miR-486.

**Dual luciferase reporter assay**

Luciferase reporters were constructed by cloning sequences from the 3' untranslated regions (3'UTRs) of the *SRSF3* mRNAs into the psiCHECK-2 vector (cat. no. C8021, Promega). Briefly, the wild-type 3' UTR (5’-GTTGGCAAAAGTGGTGTA CAGGA-3’) of the *SRSF3* gene, which contains the predicted miR-486 response element, as well as mutant 3' UTR (5’-GTTTTACAAAGTGGTTGCACTTC-3’) were synthesized and then inserted in the multiple cloning region behind the synthetic Renilla luciferase gene. HEK293 cells were co-transfected with the constructed reporter plasmid (0.05 μg) and rno-miR-486 mimics (50 nM) or the mimic negative control using Lipofectamine 3000 transfection reagent (cat. no. L3000008, Invitrogen). A reporter assay was performed 48 h after transfection using the Dual Luciferase Reporter Assay System (cat. no. E1910, Promega) according to the manufacturer’s protocol. The luciferase signal was normalized to the signal arising from an intra plasmid Renilla/firefly luciferase transfection reporter cassette in HEK293 cells. The study was conducted using three identical wells in at least three repeated experiments.

**RNA interference**

The synthesized si*SRSF3* (5'-CCCAAGAAGGAGAAGCTTT-3') and siRNA negative control (RIBOBIO, China) were transfected into CMFs using Lipofectamine RNAiMAX (cat. no. 13778150; Invitrogen) according to the manufacturer’s instructions. Briefly, the CMFs were cultured to approximately 70% confluence with complete culture medium at 37 ℃ and 5% CO_2_ in a 95% air incubator. Lipofectamine RNAiMAX was mixed with the si*SRSF3* or the siRNA negative control (100 nM), and then added into the prepared CMFs respectively. After incubation for 48 h, the treated cells were harvested for further qPCR assay, flow cytometry analysis or Western blot analysis. The study was conducted using three identical wells in at least three repeated experiments.

**RIP-qPCR**

The prepared CMFs were cultured to approximately 80% confluency, and then 2x10^6^ cells were collected for RIP-qPCR assay. An Imprint^®^ RNA Immunoprecipitation Kit (cat. no. RIP-12RXN; Sigma) was used according to the manufacturer’s instructions. Briefly, the collected cells were washed with cold PBS (pH=7.4) and centrifuged at 4 ℃ and 1600 rpm for 4 min. After the supernatant was removed, 200 μL mild lysis buffer (containing protease inhibitor and ribonuclease inhibitor) was added and mixed with the cell pellet. The mixture was centrifuged at 4 ℃ and 12000 rpm for 10 min, and then the prepared cell lysate (supernatant) was transferred to a fresh tube. Ten microliters of the prepared lysate were set as a 5% input control and stored on ice. For the preparation of beads, 40 μL protein A magnetic beads were mixed with 200 μL wash buffer in a 1.5 mL tube, and the tube was set on a magnetic stand. After the liquid phase was removed, the collected material was washed twice with 200 μL wash buffer, and the liquid phase was removed. In the following procedure, all wash steps were conducted twice with wash buffer, and the reaction tube was placed on a magnetic stand for the washing step. After the washings, the tube was removed from the magnetic stand. Then, 5 μg rabbit anti-rat IgG (whole molecule) antibody was added to the tubes, incubated at room temperature with rotation for 30 min, and washed twice with wash buffer. The bead-linked materials were resuspended in 200 μL wash buffer and then divided into two parts, in which 100 μL each was added to a new 1.5 mL tube. Then, one tube was added to 2.5 μg rat IgG, while the other was added to 2.5 μg anti-SRSF3 antibody. Both tubes were incubated at room temperature with rotation for 30 min and then washed twice with wash buffer. After the liquid phase was removed completely, the RIP reaction was conducted as follows. The prepared IgG-bead pellet and anti-SRSF3 antibody-bead pellet were resuspended in 200 μl IP buffer, and 100 μl IP buffer was added to the cell lysate. The prepared diluted cell lysate was transferred into both the prepared IgG-bead pellet mixture and anti-SRSF3 antibody-bead pellet mixture, incubated at 4 ℃ with rotation overnight, and then washed with wash buffer. The collected IgG-bead pellet mixture, anti-SRSF3 antibody-bead pellet mixture and 5% input control were applied to extract RNA using TRIzol reagent (cat. no. 15596018; Invitrogen) according to the manufacturer’s protocol. The total mRNA collected from each group was subjected to qPCR analysis, which was conducted using the following procedure: ReverTra Ace q-PCR RT Master Mix with gDNA Remover (cat. no. FSQ-301, Toyobo) was used to reverse transcribe the RNA, and the gene expression level was analyzed by SYBR Green-based real-time PCR. The reaction mixture was composed of 10 μL of 2 × SYBR Green PCR Master Mix, 1 μL of each primer, 6 μL of nuclease-free water and 2 μL of the cDNA template. Amplifications were performed on a Mini-Opticon System (Bio–Rad) using the following program: initial denaturation at 95℃ for 3 min followed by 40 cycles of amplification at 95℃ for 15 s and then 62℃ for 30 s. The relative expression was determined using the fold enrichment comparative threshold method, which is fold enrichment = 2^–△△Ct [RIP/NS]^, △△Ct [RIP/NS] = △Ct [normalized RIP] – △Ct [normalized NS], △Ct [normalized RIP or NS] = Ct [RIP or NS] – (Ct [Input] – Log_2_ [input dilution factor]) (input dilution factor = fraction of the input RNA saved). The study was conducted using three identical wells in at least three repeated experiments.

**Induction of MI and intramyocardial injection**

Two-month-old female SD rats were applied to established MI. The rats were anesthetized with ketamine (100 mg/kg, i.p.) and underwent a left intercostal thoracotomy. The left anterior descending coronary artery (LAD) was identified and then ligated directly below the left atrial appendage with 8-0-gauge nylon sutures. The presence of pallor and abnormal movement of the left ventricle confirmed LAD occlusion. Intramyocardial injections were performed within 15 min after LAD ligation. AAV9-miR-486-EGFP (titer: 10^12^ vg/mL; HANBio) was transmyocardium injected using a 10 μL Hamilton syringe. Each heart received 5 injections (10 μL/injection), with 3 injections in border areas of the ischemic zone and 2 injections in the center of the ischemic area. Same amount of AAV9-negative control (NC) was applied as control group. After injection, the chest wall was then closed, the lungs were inflated, the rat was extubated, and the thoracotomy was closed. After recovery, the rats were returned to the animal facility. The AAV9-miR-486-EGFP and AAV9-NC treated groups were evaluated for cardiac function in 8 weeks and 24 weeks after injection, and then the treated hearts were harvested for tissue proceeding. The harvested hearts were fixed with 4% paraformaldehyde, embedded in paraffin wax or Neg-50™ Frozen Section Medium (cat. no. 6502; Thermo Fisher) and sectioned. In the present study, the animal numbers used for 8-week observation were 12, 12, and 10 for the AAV9-miR-486-EGFP, AAV9-NC and sham groups. The survival rates of the AAV9-miR-486-EGFP, AAV9-NC and sham groups were 83.3% (10/12), 83.3% (10/12), and 100% (10/10). While for 24-week observation, it was 14 and 12 for the AAV9-miR-486-EGFP and AAV9-NC groups. The survival rates of AAV9-miR-486-EGFP and AAV9-NC groups were 71.4% (10/14) and 66.7% (8/12).

**Echocardiography**

Transthoracic echocardiograms were performed to analyze the cardiac function in the experimental rats. The ejection fraction (EF) was calculated using the area-length method ^20^. The experimental rats were anesthetized with ketamine (100 mg/kg, i.p.). The echocardiographic parameters were then collected using a VINNO6 echocardiogram (for 8-week treated observation; YEERAN, China) and Acuson Sequoia 256c ultrasound system equipped with a 13-MHz linear transducer from a Vevo 770 echocardiogram (for 24-week treated observation; VisualSonics, Canada). Briefly, the anterior chest wall was shaved, and the rat was placed in a left lateral decubitus position. A rectal temperature probe was inserted, and the body temperature was carefully maintained between 37 ℃ and 37.5 ℃ on a heating pad throughout the study. Parasternal long-axis, parasternal short-axis and 2 apical four-chamber views were collected in 2D-M-mode. The systolic and diastolic anatomic parameters were obtained from M-mode tracings at the mid-papillary level. In the present study, the animal numbers used for 8-week observation were 12, 12, and 10 for the AAV9-miR-486-EGFP, AAV9-NC and sham groups.

**Histological analysis**

The MI extent was analyzed at the level of the mid-papillary heart muscles and scored following Masson’s trichrome staining. The infarct size, with linear approximations to account for area gaps in histology, was expressed as a percentage of the total LV myocardial area as our and others previously described ^20,21^. The extent of MI was measured at the level of the mid-papillary heart muscles and scored following Masson’s trichrome staining. Briefly, paraffin sections were dewaxed and rinsed with water using routine protocols (frozen sections were washed with PBS directly). After iron hematoxylin staining (7 min), the sections were stained with Ponceau acid fuchsin (5 min) and rinsed with distilled water. After differentiation using a phosphomolybdic acid solution (5 min), the sections were sequentially stained with aniline blue (5 min) and 1% acetic acid (1 min). After staining, the sections were dehydrated, cleared in xylene, and then mounted with resinene. The infarct size, with linear approximations to account for area gaps in histology, was expressed as a percentage of the total LV myocardial area as our and others previously described ^20,21^. The collagen area of the infarct zone (CAIZ) was expressed as the ratio of blue staining in the infarct zone measured by Image-Pro Analyzer 6.0 software. In the present study, the animal numbers used for 8-week observation were 12, 12, and 10 for the AAV9-miR-486-EGFP, AAV9-NC and sham groups.

The area of cardiomyocytes was evaluated by Wheat Germ Agglutinin (WGA) staining, WGA was used to label the intercellular substance and cell membrane, and cardiac troponin I (cTnI) can specifically mark cardiomyocytes. The hydrated sections were pretreated with Tris-EDTA buffer mediated antigen retrieval (frozen sections washed with PBS directly). Then, the sections were incubated with WGA (cat. no. W11261; Invitrogen) for 10 min and washed with PBS. The sections were blocked with 1% BSA at room temperature for 1 h, then incubated with anti-cTnI (1:100; cat. no. ab47003; Abcam) at 4 ℃ overnight. After washing, the sections were incubated with the donkey anti-rabbit Alexa-Fluor 555 (1:1000) for 1 h and then incubated with Hoechst for 15 min. The sections were sealed with Antifade Mounting Medium and observed under a microscope. The cross-sectioned area of cardiomyocytes in the infarct zone and border zone were measured by Image-Pro Analyzer 6.0 software. In the present study, the animal numbers used for 8-week observation were 10 and 8 for the AAV9-miR-486-EGFP, AAV9-NC groups, while it was 10 and 10 for 24-week observation for the AAV9-miR-486-EGFP, AAV9-NC groups.

**Immunohistochemistry staining**

The immunostaining for vWF, a marker of endothelial cells, was applied to measure small blood vessel density in the infarct zone and border zone. The sections were dewaxed and rehydrated (frozen sections washed with PBS directly), then blocked with 1% BSA at room temperature for 1 h. The sections were incubated with anti-vWF (1:300; cat. no. F3520; Sigma) at 4 ℃ overnight. After washing, the sections were sequentially incubated with peroxidase-conjugated goat anti-rabbit IgG (cat. no. SA00001-2; Proteintech) for 1 h and then DAB-Plus Staining Kit (cat. no. 00-2020; Life Technologies). After staining, the sections were dehydrated, cleared in xylene, and then mounted with resinene. The vWF^+^ blood vessels present throughout the infarct zone and border zone were photographed and counted under a microscope. The number of vessels per mm^2^ was compared among the different groups. In the present study, the animal numbers used for 8-week observation were 6 and 5 for the AAV9-miR-486-EGFP, AAV9-NC groups, while it was 10 and 8 for 24-week observation for the AAV9-miR-486-EGFP, AAV9-NC groups.

**Treadmill test**

A treadmill (SA101C, Sansbio) was used to measure the endurance and physical fitness of AAV9-miR-486-EGFP (n=10)- and AAV9-NC (n=8)-treated MI rats 24 weeks after MI. The treated rats were trained by a motorized treadmill using the moderate training protocol, which was set at a slope of 5°, speed of 18 m/min, and acceleration of 70 m/min^2^. The running distance and time were recorded when the rat reached exhaustion (failed to run).

**Statistical analysis**

An independent samples t-test was performed using GraphPad prism software to determine the P-values in repeated experiments. All values are expressed as the mean ± standard deviation (S.Dev). *p*<0.05 was set as statistically significant differences.
